# Ozone mitigates extended growing season and enhanced vegetation greenness driven by environmental change

**DOI:** 10.1038/s41467-026-71959-y

**Published:** 2026-04-20

**Authors:** Hao Yin, Lin Meng, Andrew D. Richardson, Maria Val Martin, Jiafu Mao, Huidong Li, Jonathan M. Gilligan, Hemraj Bhattarai, Amos P. K. Tai

**Affiliations:** 1 Department of Earth and Environmental Science, Vanderbilt University, Nashville, TN USA; 2 Center for Ecosystem Science and Society, Northern Arizona University, Flagstaff, AZ USA; 3 School of Informatics, Computing, and Cyber Systems, Northern Arizona University, Flagstaff, AZ USA; 4 Leverhulme Centre for Climate Change Mitigation, School of Biosciences, University of Sheffield, Sheffield, UK; 5 Environmental Sciences Division, Oak Ridge National Laboratory, Oak Ridge, TN USA; 6 Division of Environment and Sustainability, The Hong Kong University of Science and Technology, Hong Kong, China; 7 Department of Earth and Environmental Sciences, The Chinese University of Hong Kong, Hong Kong, China; 8 State Key Laboratory of Agrobiotechnology, and Institute of Environment, Energy and Sustainability, The Chinese University of Hong Kong, Hong Kong, China

**Keywords:** Phenology, Ecosystem ecology, Ecosystem ecology

## Abstract

Rising temperature and elevated CO_2_ concentrations lead to extended growing seasons and enhanced vegetation greenness in terrestrial ecosystems, especially across the Northern Hemisphere. However, whether and to what extent surface ozone, an anthropogenic environmental factor, affects vegetation phenology and greenness remains unexplored at a large scale. Integrating ground-based ozone observations with multiple satellite observations, we demonstrate that surface ozone significantly shortened the growing season by delaying start of season and advancing end of season. Additionally, ozone reduced growing-season vegetation greenness, as reflected in decreased annual accumulated Enhanced Vegetation Index and maximum Enhanced Vegetation Index. These impacts show pronounced spatial heterogeneity, varying in magnitude across the United States, Europe, and China over the past decade, highlighting ozone’s diverse impact on vegetation across regions. Our study predicts that continuously increasing surface ozone concentration will mitigate warming-driven lengthening the growing season by 2 to 4 days, reduce maximum Enhanced Vegetation Index by 0.4% to 8.3%, and reduce annual accumulated Enhanced Vegetation Index by 1.0% to 5.6% in 2050 under Shared Socioeconomic Pathway 5-8.5 scenario. Our findings highlight the imperative need for strategic surface ozone regulation to optimize vegetation health and maximize the capacity for carbon sequestration.

## Abstract (web summary)

Warming and rising CO₂ generally promote vegetation growth, but the role of surface ozone remains unclear. This study suggests that ozone shortens growing seasons and reduces greenness, offsetting climate-driven gains.

## Introduction

Rising temperatures and elevated carbon dioxide (CO_2_) levels alter vegetation canopy dynamics and carbon uptake in the terrestrial ecosystem^1,2^. An increase in temperature causes an earlier start of season (SOS) by 7–9 days, and a later end of season (EOS) by 5–8 days over the Northern Hemisphere, resulting in a prolonged growing period^3,4^. Rising atmospheric CO_2_ levels and warming increase vegetation greenness, as indicated by integrated annual enhanced vegetation index (EVI) and peak EVI^5,6^. These changes in vegetation seasonality and canopy density influence the energy budget, biogeochemical cycles, and feedback mechanisms of climate system. In addition, these ecosystem changes also enhance the terrestrial carbon sink, serving as key considerations in the development and implementation of carbon-neutral policies^7,8^. However, the influence of anthropogenic factors beyond temperature and CO_2_, notably surface ozone, on the timing (i.e., phenology) and magnitude (i.e., greenness level) of vegetation canopy changes remains relatively unexplored at large scales, leading to large biases in predicting future ecosystem functioning.

Surface ozone, a secondary pollutant formed from the reaction of precursor species (e.g., carbon monoxide, volatile organic compounds, and nitrogen oxides) in the presence of sunlight, has long been recognized as a harmful air pollutant with detrimental effects on vegetation^9,10^. Human activities, including the emissions of pollutants from industrial processes and transportation, along with environmental factors like rising temperatures, drought, and wildfires, are identified as crucial drivers of increasing surface ozone concentrations^11–14^. In developed regions like the USA and Europe, policies aimed at reducing ozone precursors led to a gradual decline in surface ozone levels of −0.81 and −0.30 ppbv/year, respectively, over the past decades^15,16^. In contrast, many developing countries, particularly those undergoing rapid industrialization and urbanization, experienced rising surface ozone levels (e.g., by 2.4 ppbv/year in China), which poses significant challenges for ecosystem health^17,18^. These contrasting ozone trends, which interact with environmental change, may imply diverging effects on ecological processes and functions in different regions.

Beyond visible ozone injury on individual species, the impacts of surface ozone on vegetation phenology and growth are observed primarily at the site level, using field observation or greenhouse experiments, or at the ecosystem scale through modeling approaches. For example, ozone pollution is found to delay spring phenology by a week for birch, horse chestnut, and *Populus*^19,20^. Ozone exposure reduces photosynthetic rates and accelerates leaf senescence, thereby reducing ecosystem productivity and carbon sink^21–24^. However, the extent to which reduced ecosystem productivity is attributed to declines in canopy greenness, as well as the regional variations in ozone’s effects on vegetation phenology and greenness, remains largely unknown. Consequently, current ecosystem predictions often neglect the potential impact of varying surface ozone concentrations in the future, which depends on the implementation and effectiveness of policies. This oversight limits current carbon policies by failing to account for ozone-induced reductions in carbon sequestration. A more integrated approach that considers ozone, temperature, and CO₂ effects on vegetation is essential for accurately predicting ecosystem changes.

**Fig. 1:**
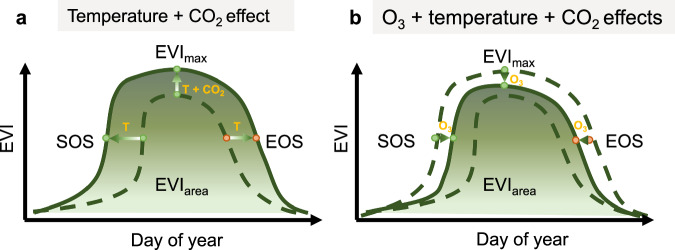
Schematic representation of ozone, temperature, and CO_2_ effects on vegetation phenology and greenness. **a** Increasing temperatures (T) advance start of season (SOS) and delay end of season (EOS), while both elevated T and CO_2_ concentrations increase vegetation greenness, i.e., the maximum EVI (EVI_max_) and cumulative EVI throughout the growing season (EVI_area_). **b** Increased surface ozone concentration (O_3_) offsets the influence of CO_2_ and T by delaying SOS, advancing EOS, and diminishing EVI_max_ and EVI_area_ during the growing season.

In this study, we focus on the following questions: (a) Is there a detectable effect of ozone on vegetation phenology and greenness at a regional scale? (b) What are the magnitudes of ozone, temperature, and CO_2_ effects on vegetation phenology and greenness? (c) How will vegetation phenology and greenness change under future climate and ozone scenarios? We hypothesize higher ozone concentrations offset the lengthened growing season and enhanced vegetation greenness by increasing temperature and CO_2_ levels (Fig. 1). To test this hypothesis, we investigate four key satellite-derived vegetation indicators: SOS and EOS to represent phenology, and maximum EVI (EVI_max_) and cumulative EVI throughout the entire growing season (EVI_area_) to represent greenness, using the NASA Visible Infrared Imaging Radiometer Suite (VIIRS) Global Land Surface Phenology (GLSP) data product. We quantify ozone exposure using Accumulated dose of ozone over a Threshold of 40 ppbv (AOT40) and average surface ozone concentration derived from ground-based observation networks, most of which are located in urban areas. We assess the effects of ozone, temperature, and CO_2_ on the four vegetation indicators for the USA, Europe, and China using partial correlation analysis (see “Methods”). Future changes in vegetation phenology and greenness by 2050 are projected using an integrated machine-learning model (LightGBM and SHapley Additive exPlanations (SHAP)), driven by climate projections from the sixth phase of the Coupled Model Intercomparison Project (CMIP6) and surface ozone data from the Community Earth System Model version 2 (CESM2). Because most monitoring sites are in urban areas, our estimates of ozone effects primarily capture responses of urban vegetation, while still providing insight into ozone–phenology relationships across broader land cover types.

## Results

### Significant ozone effect on vegetation phenology and greenness across regions

**Fig. 2:**
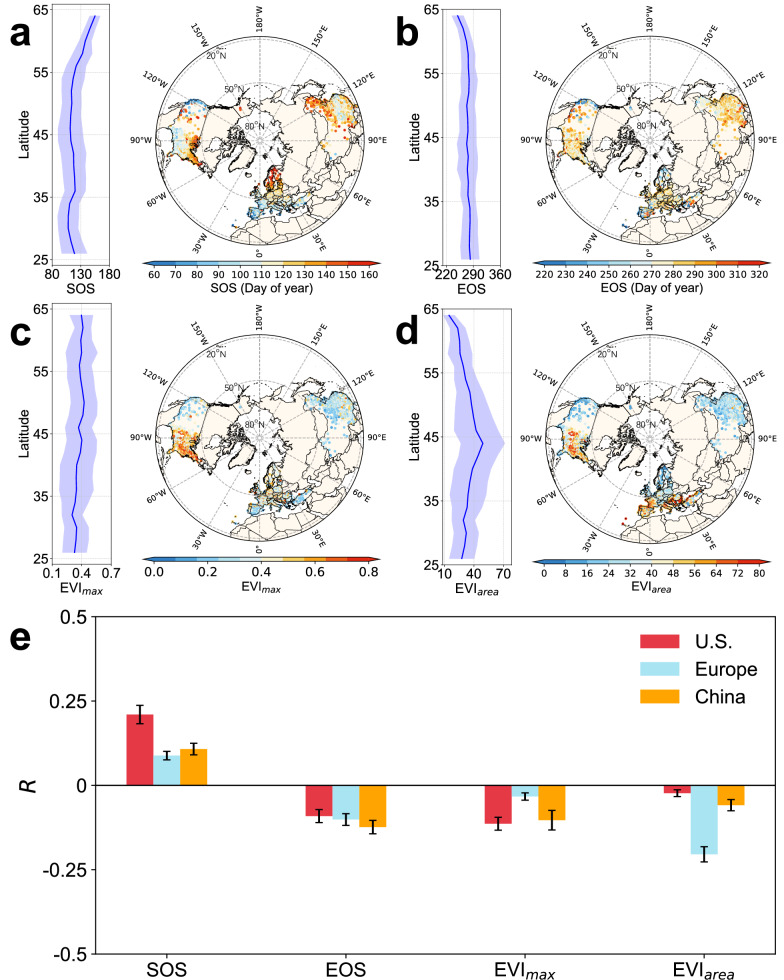
Significant ozone effect on vegetation phenology and greenness in the USA, Europe, and China. **a–d** spatial patterns of long-term averaged start of season (SOS) (**a**), end of season (EOS) (**b**), maximum EVI (EVI_max_) (**c**), and cumulative EVI throughout the growing season (EVI_area_) (**d**), along with their latitudinal variation. The line indicates the average of each variable and shadow indicates the standard variances. **e** Partial correlation coefficients (*R*) between Accumulated dose of ozone Over a Threshold of 40 ppbv (AOT40) and SOS, EOS, EVI_max_, and EVI_area_, respectively, for USA (2013–2022, *n* = 3753), Europe (2013–2022, *n* = 11401), and China (2015–2022, *n* = 9272). Bars represent mean values, and error bars represent the standard deviations across years.

Both vegetation indicators and environmental drivers exhibit pronounced regional variation across China, the USA, and Europe. China experiences the highest average annual AOT40 (35.95 × 10^3^ ppbv·hour), considerably exceeding levels in the USA (14.38 × 10^3^ ppbv·hour) and Europe (9.61 × 10^3^ ppbv·hour) (Fig. S1), with pronounced seasonal variations peaked in summer and autumn (Fig. S2). Decadal mean AOT40 show a distinct east low-west high spatial pattern in the USA, primarily attributed to the transport of ozone pollution originating from East Asia and stratospheric ozone intrusion^25–27^. Ozone levels show similar spatial patterns with AOT40 (Fig. S3). The SOS shows clear latitudinal patterns, i.e., delayed with increasing latitude, especially in Europe where a difference of 9–12 days in SOS is found in northern countries compared to the Mediterranean regions (Fig. 2a). In contrast, regional patterns in SOS across the USA and China reflect localized climatic influences; the western USA exhibits an earlier SOS than the east, while southeastern China shows a delayed SOS due to the spring East Asian monsoon, which brings heavy rainfall and reduced solar radiation (25). The EOS, however, exhibits less distinct latitudinal patterns in all three study regions (Fig. 2b). Greenness indicators, EVI_max_ and EVI_area_, similarly show notable regional contrasts, e.g., higher in the eastern USA compared to the west (Fig. 2c, d). Temperature patterns align closely with SOS, showing clear latitudinal variations (Fig. S4). Average annual CO_2_ levels are very similar across China (407 ppbv), Europe (406 ppbv), and the USA (405 ppbv), with no discernable latitudinal pattern (Fig. S5).

We found ozone has significant effects on both vegetation phenology and greenness across all three study regions (USA, Europe, and China), after removing effects of temperature, vapor pressure deficit (VPD), precipitation, and CO_2_ through spatial partial correlation analysis (Figs. 2e and S6, *p* < 0.05). Specifically, higher temperature is associated with earlier SOS and later EOS, lengthening of the growing season, and increased EVI_max_ and EVI_area_, enhancing vegetation greenness (Fig. S6b, *p* < 0.05), while higher CO_2_ levels is associated with a further increase in EVI_max_ and EVI_area_ (Fig. S6e, *p* < 0.05), consistent with the findings of existing studies^28–30^. In contrast, higher AOT40 is associated with delayed SOS (partial correlation coefficient between AOT40 and SOS [R_AOT40-SOS_]: 0.21 ± 0.03 in the USA (Mean ± Standard Deviation [S.D.]), 0.09 ± 0.01 in Europe, and 0.11 ± 0.02 in China), and advanced EOS (R_AOT40-EOS_: −0.09 ± 0.02, −0.10 ± 0.02, and −0.12 ± 0.02) across all three regions (Fig. 2e). In other words, elevated ozone is associated with the shortening of growing season by shifting both SOS and EOS. Moreover, higher AOT40 is associated with reduced EVI_max_ (R_AOT40-EVImax_: −0.11 ± 0.02, −0.03 ± 0.01, and −0.10 ± 0.02) and EVI_area_ (R_AOT40-EVIarea_: −0.03 ± 0.01, −0.20 ± 0.02, and −0.06 ± 0.02). The largest ozone impact on SOS and EVI_max_ occurred in the USA, the greatest effect on EOS was in China, and the largest effect on EVI_area_ was found in Europe. We also conducted similar partial correlation analyses using average ozone concentrations (instead of AOT40, Fig. S6), an alternative XCO_2_ dataset (GOSAT Global Land 1° Mapping, see “Methods”; Fig. S7), and MODIS phenology data (instead of VIIRS, Fig. S8), all of which yielded consistent results. Analyses across land cover types showed similar patterns, though with varying effect magnitudes (Figs. S9 and S10).

### Stronger ozone effect on EOS than SOS over years

**Fig. 3:**
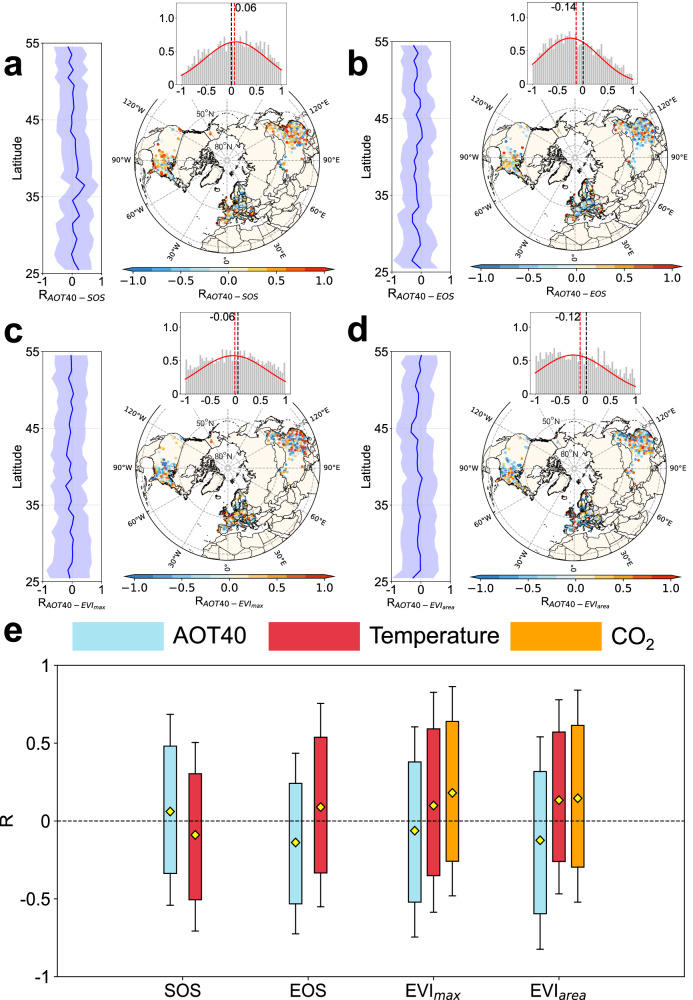
The effects of ozone, temperature, and CO_2_ on vegetation phenology and greenness over time. **a–d** Spatial patterns of temporal partial correlation coefficients (*R*) between Accumulated dose of ozone Over a Threshold of 40 ppbv (AOT40) and start of season (SOS) (**a**), end of season (EOS) (**b**), maximum EVI (EVI_max_) (**c**), and cumulative EVI throughout the growing season (EVI_area_) (**d**). The inserted subplots above the map show histograms of *R*. Black dashed lines indicate zero values, while red dashed lines and numbers represent the mean coefficients. Left subplots show the changes with latitude, with lines indicate the averaged *R* and shadow indicates the standard variances. **e** Partial correlation coefficients between AOT40, temperature, CO_2_ and vegetation phenology and greenness (*n* = 24426). The diamonds indicate the average of partial correlations; box edges denote 25 and 75% quartiles; the whiskers represent 25% of the standard deviation. Significance was assessed using two-sided tests. The spatial patterns of statistical significance at the 95% confidence level (*p* < 0.05) over each site are presented in Figs. S19–S22.

To assess temporal relationships, we calculated the temporal partial correlation coefficients of four vegetation indices with environmental factors for each site (Fig. 3). We found the effects of ozone, temperature, and CO_2_ on vegetation phenology and greenness are temporally and spatially consistent, although temporal effects show larger variations across sites. In general, the temporal R_AOT40-SOS_ is positive (0.06 ± 0.004 on average, Mean ± Standard Error [S.E.]), indicating increasing AOT40 is associated with delay in SOS over years, although the correlation is negative in Northwest China, Western USA, and Northern Europe (Fig. 3a and e). In contrast, the temporal R_AOT40-EOS_ is generally negative (−0.14 ± 0.003), which indicates increasing AOT40 is associated with advancing EOS over years (Fig. 3b and e). The negative R_AOT40-EVImax_ (−0.06 ± 0.004) and R_AOT40-EVIarea_ (−0.12 ± 0.004) indicate that increasing AOT40 is associated with decreasing vegetation greenness (Fig. 3c, d). Interestingly, we found the average ozone effect (R_AOT40-SOS_: 0.06 ± 0.004) is weaker than the average temperature effect (R_T-SOS_: −0.09 ± 0.004) on SOS, but stronger than the temperature effect on EOS (R_AOT40-EOS_: −0.14 ± 0.003, and R_T-EOS_: 0.09 ± 0.004) (Figs. 3e and S11), indicating that ozone plays a comparatively greater role in EOS than in SOS. For EVI_max_ and EVI_area_, CO_2_ shows the strongest correlations (R_CO2-EVImax_: 0.18 ± 0.004, R_CO2-EVIarea_: 0.15 ± 0.004), followed by AOT40 (R_AOT40-EVImax_: −0.06 ± 0.004, R_AOT40-EVIarea_: −0.12 ± 0.004), while the effect of temperature is comparatively modest (R_T-EVImax_: 0.10 ± 0.004, R_T-EVIarea_: 0.13 ± 0.004) (Figs. 3e and S18). We conducted the same temporal partial correlation analysis using ozone concentration, instead of AOT40, and obtained similar results (Fig. S12).

The long-term trends of ozone, temperature, and CO_2_ as well as their impact on vegetation indexes vary widely across the three study regions (Figs. S13–S18). Temperature and CO_2_ increased in similar way across three regions, but ozone increased rapid in China, while decreased in the USA and Europe (Figs. S13–S16). In China, the correlation of AOT40 on delaying SOS (R_AOT40-SOS_: 0.11 ± 0.003) and advancing EOS (R_AOT40-EOS_: −0.19 ± 0.002) is greater than the correlation of temperature (R_T-SOS_: −0.08 ± 0.001, R_T-EOS_: 0.03 ± 0.002) (Fig. S11), suggesting the profound role of ozone on vegetation in China. In contrast, in both USA and Europe, AOT40 correlation with vegetation phenology (USA: R_AOT40-SOS_: 0.16 ± 0.004 and R_AOT40-EOS_: −0.06 ± 0.003; Europe: R_AOT40-SOS_: −0.04 ± 0.003 and R_AOT40-EOS_: −0.03 ± 0.004) is weaker than temperature correlation (R_T-SOS_: −0.21 ± 0.004 and R_T-EOS_: 0.17 ± 0.003 for USA; R_T-SOS_: −0.14 ± 0.004 and R_T-EOS_: 0.11 ± 0.003 for Europe) (Figs. S11 and 17). In Europe, AOT40 effect on EVI_max_ (R_AOT40-EVIarea_: −0.17 ± 0.003) is more pronounced compared to the CO_2_ effect (R_CO2-EVIarea_: 0.05 ± 0.004), while the opposite is observed in China and the USA (Figs. S11 and S18).

### Vegetation changes under future ozone scenarios

**Fig. 4:**
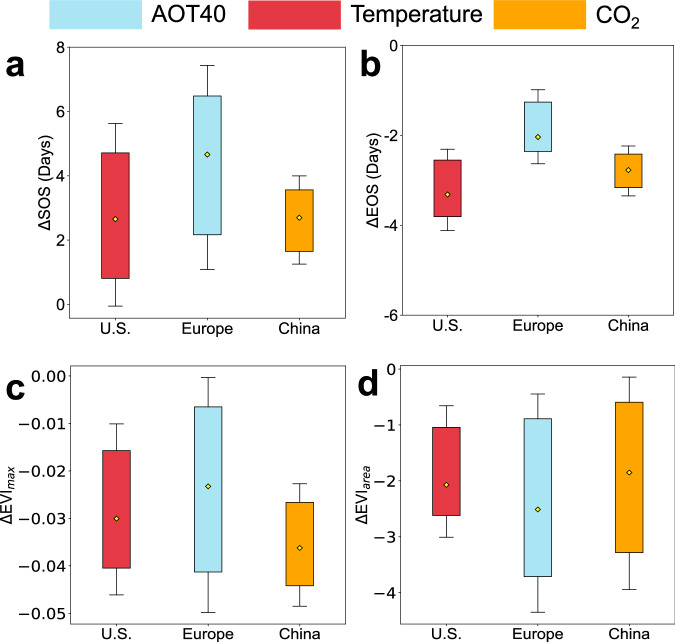
Predicted ozone impact on vegetation phenology and greenness in 2050 in the USA, Europe, and China. **a**–**d** Differences in start of season (SOS) (**a**), end of season (EOS) (**b**), maximum EVI (EVI_max_) (**c**), and cumulative EVI throughout the growing season (EVI_area_) (**d**) derived from predictions with and without ozone effects using CESM2 data under Shared Socioeconomic Pathway (SSP) 5–8.5 (see “Methods”) in the USA (*n* = 121), Europe (*n* = 528), and China (*n* = 575). The diamonds indicate the mean values; box edges denote 25 and 75% quartiles, and the whiskers represent 25% of the standard deviation.

To assess the potential impacts of future climate and ozone changes on vegetation, we developed a machine-learning model (LightGBM with SHAP interpretation) to project changes in four vegetation indicators by 2050 under climate scenarios with either fixed 2022 ozone (no ozone effect) or CESM2 ozone projections. The difference between the two simulations represents the impact of ozone on future vegetation changes. We found the continuous increase in surface ozone concentration is expected to diminish the effects of warming and elevated CO_2_ on lengthening the growing season and enhancing vegetation greenness for 2050 (Fig. 4). Specifically, under fixed ozone scenarios (no ozone effect), SOS will advance by 5–19 days by 2050, EOS will delay by 10–32 days, EVI_max_ will increase by 0.15–0.45, and EVI_area_ will increase by 23–47 by 2050 (Fig. S24). After considering ozone effect in the prediction, SOS delays by about 3, 5, and 3 days in the USA, Europe, and China, respectively (SHAP: 0.29 ± 0.18, mean ± S.D.), EOS advances by 3, 2, and 3 days (SHAP: −0.21 ± 0.15), EVI_max_ decreased by 0.03, 0.02, and 0.04 (SHAP: −0.20 ± 0.19), while EVI_area_ decreased by 2.07, 2.51, and 1.85 (SHAP: 0.16 ± 0.12, Figs. 4 and S25). In other words, increasing surface ozone concentration will mitigate warming-driven lengthening the growing season by 2 to 4 days, reduce maximum EVI by 0.4–8.3%, and reduce annual accumulated EVI by 1.0–5.6% in 2050 under Shared Socioeconomic Pathway (SSP) 5–8.5. These findings align with the results obtained through partial correlation analysis of historical patterns. This consistency between different methodological approaches enhances the reliability of our conclusions regarding impacts of ozone.

## Discussion

This study highlights ozone as a critical factor that shortens the growing season of vegetation and reduces vegetation greenness worldwide, especially in urban areas. While vegetation growing length and greenness are generally expected to increase with increases in temperature and CO_2_ levels in the near future^31,32^, the predicted rise in ozone levels is likely to offset these gains by reducing the duration of the growing season and vegetation greenness, thereby diminishing carbon sequestration capacity of ecosystems. Although the magnitude of the ozone effect is moderate compared to temperature and CO₂, incorporating it into future projections improves the accuracy of predicting vegetation dynamics and ecosystem functioning. Mitigating ozone pollution has profound and far-reaching significance for sustaining ecosystem health, guiding effective environmental policy, and supporting global sustainability goals.

Previous work has shown that ozone affects plant phenology and vegetation greenness through a multifaceted process^33,34^. As a powerful oxidant, ozone infiltrates plant tissues and disrupts cellular functions, impairing photosynthesis, and altering hormonal regulation^35,36^. Stomatal uptake serves as the primary pathway for ozone entry into leaves, where it rapidly decomposes and generates reactive oxygen species (ROS), further intensifying oxidative stress^37,38^. This stress reduces the activity of key photosynthetic enzymes such as Rubisco and disrupts chloroplast ultrastructure, thereby diminishing photosynthetic capacity^39,40^. Ozone-induced oxidative stress triggers the formation of ROS within plant cells, leading to cellular damage and a compromised defense mechanism against environmental stressors^41,42^. These physiological disruptions, combined with the inhibition of carbon assimilation, contribute to premature senescence (earlier EOS) and delayed flowering, consequently shortening the growing season^20,43,44^. As the deleterious effects of ozone-induced oxidative stress accumulate within plant cells, chlorophyll content and the consequent greenness decrease^45,46^. This diminished capacity for photosynthesis results in reduced biomass production and overall growth, impacting vegetation greenness^47,48^.

Ozone-induced damage varies widely across species, reflecting differences in antioxidant capacity, stomatal behavior, and repair mechanisms^49^. Species-specific responses, for example, shifts in carbohydrate metabolism in *Phoebe spp*. or photosynthetic decline in *Fagus crenata* and *Quercus ilex*, are further modulated by nutrient availability and interacting stressors such as drought and heat, which can either mitigate or amplify ozone injury^50,51^. These context-dependent responses highlight the need for models that capture species traits and multi-stressor interactions to better predict ecosystem-level impacts of ozone under future scenarios^52^.

The divergent temporal trends observed in ozone concentration between China, on the one hand, and the USA and Europe, on the other, reflect differences in environmental regulation. USA and Europe have developed and implemented ozone pollution standards and preventive through the Clean Air Act Extension by the USA in 1970^53^, and the Convention on Long-range Transboundary Air Pollution by Europe in 1979^54^. More importantly, these regions have consistently updated standards of air quality and emissions controls, with cross-departmental and regional cooperation for coordinated control measures targeting multiple pollutants, especially NO_x_ and VOCs—key ozone precursors^55–57^. Consequently, most parts of the USA and Europe have curbed ozone levels and maintained air quality within prescribed limits in recent years. In contrast, China’s rapid industrialization and vehicle emissions drove elevated ozone levels—around 5%/year from 2013 to 2019^14,17,18^, as early policies focused mainly on NO_x_, SO_2_, and PM_2.5_. More recently, China has initiated measures aimed at curtailing the emissions of ozone precursors through coordinated control of VOCs, NO_x_, and PM_2.5_, with a target to reduce NO_x_ and VOCs by 10% in 2025 compared with 2020^14^.

We found that ozone impacts are highly spatially heterogeneous. Regional differences arise from variation in vegetation composition, meteorology, and chemical environments. In hot, high-VPD regions such as parts of China, reduced stomatal conductance may limit ozone uptake, yet concurrent high ozone and temperature can still accelerate senescence and reduce assimilation, as shown in this study. Urbanization intensity is also a key factor, with stronger ozone formation observed in dense city clusters such as the Pearl River and Yangtze River Deltas compared to surrounding rural areas. In addition, co-occurring pollutants like PM_2.5_ alter light and radiation regimes, further compounding ozone effects and contributing to regional variability in phenology and productivity. These complexities underscore the need for region-specific strategies that account for both ozone levels and local ecological contexts when aiming to sustain ecosystem function.

Future strategies for ozone pollution control require strengthened cooperation within regional and international frameworks. Due to its relatively long atmospheric lifetime, ozone has the capacity to travel long distances, contributing to cross-boundary ozone pollution. For example, the western USA has struggled to meet established ozone standards, potentially because of the long-range transport of ozone and its precursors from other regions^25,27,58^. Changes in ozone pollution will also be affected by social and economic factors. The transition toward vehicle electrification and renewable electricity generation promises to significantly reduce NO_x_ emissions, especially in urban areas^59^. Additionally, in many rural areas, agricultural soil emissions of NO_x_ are a major contributor to ozone, so policy responses will need to look beyond energy and transportation^60–62^. In addition to mitigating the impact of urban ozone pollution, adopting a forward-looking approach involves cultivation of plant species that exhibit lower sensitivity to ozone, such as *Ginkgo biloba* and *Pinus armandii*^63^.

Monitoring surface ozone levels worldwide remains a significant challenge. The integration of statistical modeling, remote sensing observations, and process modeling of transport and atmospheric chemistry have shown considerable advancement in estimating high-resolution spatial patterns of surface ozone concentration from site observations^64,65^. However, current monitoring stations are predominantly situated in urban areas^66^. Given that ozone concentration is affected by not only anthropogenic emissions but also geographical features, vegetation types, meteorological conditions, and atmospheric transport processes^67^, representative ozone monitoring stations in rural and forested areas should be prioritized. At the same time, satellite instruments such as TROPOMI now provide global, repeated coverage of tropospheric ozone, enabling the detection of broad spatial and temporal patterns that cannot be resolved by sparse ground networks alone. Although satellite retrievals have limitations and require careful validation, fusing satellite products with ground-calibrated observations offers a promising pathway for spatial extrapolation of ozone estimates beyond urban cores. Similar data fusion approaches have been successfully applied in eddy covariance flux studies to extend site level measurements to regional scales, and they are increasingly used to track ozone and smoke from remote fires^68^.

Our findings imply considering the ozone effect on vegetation growth in Earth system models is crucial for accurately predicting future changes in ecosystem dynamics and terrestrial carbon sequestration^34,69^. For example, most current phenology models mainly consider temperature and light availability^63^, overlooking the impacts of ozone and other pollutants on phenology and canopy greenness. This omission is significant, as longer growing seasons and higher greenness are associated with greater cumulative CO₂ assimilation, yet our results show that ozone shortens the season and reduces greenness, reducing seasonal carbon uptake^70^. Current model frameworks, such as Community Land Model (CLM), lack explicit ozone-phenology mechanisms, resulting in systematic biases in simulating vegetation productivity and carbon exchange. Additionally, the duration of O_3_ exposure varies across vegetation types, e.g., evergreen species may experience longer O_3_ exposure periods compared to deciduous species, potentially leading to greater cumulative damage. Future studies should account for these differences when refining models across spatially heterogeneous regions to improve their completeness and accuracy.

Several uncertainties remain in our study. Our results may underestimate the impact of ozone because AOT40 does not accurately reflect the full extent of ozone exposure, and plants often respond at lower ozone concentrations. A more process-based approach is to consider ozone uptake fluxes (i.e., stomatal ozone uptake at the leaf and canopy scale), which better reflect the actual dose received by vegetation. However, the lack of large-scale flux-based ozone observations highlights the need to establish more flux sites across different land covers. Beyond the drivers analyzed here, factors such as nitrogen deposition, PM_2.5_, and co-pollutants may also influence phenology, either directly or through interactions with ozone^71^. For instance, nitrogen deposition may alter plant sensitivity to ozone by modifying photosynthetic capacity and nutrient availability, while PM_2.5_ can affect light availability and radiation diffusion^72^. These limitations highlight the importance of developing more comprehensive, integrated assessments as broader and higher-resolution pollution datasets become available.

In summary, we report significant effects of ozone on vegetation phenology and greenness, i.e., high concentrations of ozone delay SOS and advance EOS, shortening the growing season, and reduce vegetation greenness, potentially decreasing the carbon sequestration of ecosystem. By predicting future vegetation phenology and greenness under climate and ozone scenarios, we emphasize that ozone is expected to partly mitigate the increase in vegetation greenness driven by warming and elevated CO_2_. These findings provide insights into the impact of ozone pollution on vegetation health and have profound implications for the development of vegetation models, carbon cycle simulations, and carbon neutrality policies. Understanding the ozone effect will better inform policy making on ozone regulation toward ecosystem health and carbon neutrality, thereby fostering a more sustainable and environmentally resilient future.

## Methods

### Ground-based ozone observations

Ground-based ozone observations in China, Europe, and the USA are available from the China National Environmental Monitoring Centre (CNEMC) network (http://www.cnemc.cn/en/), the European Environment Agency (EEA, https://www.eea.europa.eu/), and the United States Environmental Protection Agency (https://www.epa.gov/outdoor-air-quality-data/download-daily-data), respectively. These datasets have been widely used in numerous studies to assess the situation of air pollution under climate change worldwide^73^. These ground-level ozone data are measured using active differential absorption ultraviolet (UV) analyzers. To ensure the data quality, we remove unreliable outliers following previous studies^14,74^ in the following steps: (a) convert all hourly measurement data into Z scores, (b) remove the data if (1) Z_i_ is larger or smaller than the previous value Z_i-1_ by 9 (|Z_i_–Z_i-1_| >9); or (2) the absolute value of Z_i_ is greater than 4 (|Z_i_| >4); or (3) the ratio of Z_i_ to the third-order center moving average is greater than 2 ($\frac{3{Z}_{i}}{{Z}_{i-1}+{Z}_{i}+{Z}_{i+1}} > 2$), where i represents the i-th hourly measurement data. We selected ozone sites in the following land cover types: evergreen needleleaf forests, evergreen broadleaf forests, deciduous needleleaf forests, deciduous broadleaf forests, mixed forests, shrublands, savannas, and urban lands. The land cover type data is extracted from MODIS Land Cover Type (MCD12Q1) Version 6.1 data product (https://lpdaac.usgs.gov/products/mcd12q1v061/). The final dataset includes hourly ozone data in China from 2015 to 2022 and in Europe, and the USA from 2013 to 2022.

### Vegetation dataset

We extracted vegetation phenology and greenness observations at the ozone sites in the USA, Europe, and China from the NASA VIIRS GLSP data product (https://lpdaac.usgs.gov/products/vnp22q2v001/). The VIIRS GLSP product generated phenological metrics of individual vegetation growing cycles on the vegetated land surface at a spatial resolution of 500 m across the globe^75^. In this study, we selected four indicators: Date_Mid_Greenup_Phase (the date at a mid-greenup phase, SOS) Date_Mid_Senescence_Phase (the date at a mid-senescence phase, EOS), EVI2_Onset_Greenness_Maximum (the EVI2 value at maturity onset, EVI_max_), and EVI2_Growing_Season_Area (the integrated EVI2 during a growing season, EVI_area_). We used VIIRS data from 2015 to 2022 in China and from 2013 to 2022 in Europe and the USA, corresponding to the ozone data time frame.

To test the robustness of the results, we also use data from the Terra and Aqua combined Moderate Resolution Imaging Spectroradiometer (MODIS) Land Cover Dynamics (MCD12Q2) Version 6.1 data product for 2013–2022. The MCD12Q2 Version 6.1 data product is derived from time series of the 2-band Enhanced Vegetation Index (EVI2) calculated from MODIS Nadir Bidirectional Reflectance Distribution Function (BRDF)-Adjusted Reflectance (NBAR). Vegetation phenology metrics at 500 m spatial resolution are identified for up to two detected growing cycles per year. We used the variable “Greenup_1” as SOS, the variable “MidGreenup_1” as EOS, the variable “EVI_Area_1” as EVI_area_. For EVI_max_, we used the variable “EVI_Minimum_1” add the variable “ EVI_Amplitude_1”.

### Meteorology datasets

The meteorological dataset from 2013 to 2022 at the ozone sites were obtained from the latest (v5) ERA5-Land climate reanalysis hourly dataset from the European Center for Medium-Term Weather Forecasting (ECMWF). Produced by an advanced four-dimensional variational (4D-Var) assimilation system, ERA5-Land reanalysis dataset provides global land meteorological data at 0.1° resolution (https://climate.copernicus.eu/climate-reanalysis)^76^. To enable the ERA5-Land operation computationally affordably, the forcing process is not directly coupled to the atmospheric model, instead it is generated by running the ECMWF land surface model driven by the downscaled ERA5 climate reanalysis in conjunction with an additional lapse-rate correction. In this study, we extracted 2-m temperature, surface precipitation, and surface relative humidity. VPD is a critical metric, representing the difference between the saturation vapor pressure (air’s maximum water-holding capacity) and the actual vapor pressure (current water vapor content), which quantifies the atmospheric demand for water evaporation and influences plant transpiration, ecosystem productivity, and climate studies. The VPD is calculated as:

1 $${VPD}={e}_{{sat}}-{e}_{{actual}}$$

Where ${e}_{{sat}}$ is the saturation vapor pressure, computed using the Magnus formula:

2 $${e}_{{sat}}=0.6108\times \exp \left(\frac{17.27\times T}{T+237.3}\right)$$

and ${e}_{{actual}}$ is the actual vapor pressure, derived from relative humidity (RH):

3 $${e}_{{actual}}=\frac{{RH}}{100}\times {e}_{{sat}}$$

### Carbon dioxide (CO_2_) datasets

We used two historical XCO_2_ datasets, SNDRAQIL3CMCCP and Global land 1° Mapping-XCO_2_, to account for the variability in atmospheric CO_2_. SNDRAQIL3CMCCP dataset was obtained by Sounder SIPS: AQUA AIRS IR-only Level 3 CLIMCAPS product (https://disc.gsfc.nasa.gov/datasets/SNDRAQIL3CMCCP_2/summary), which is retrieved by AIRS (Atmospheric Infrared Sounder) with the CLIMCAPS (Community Long-term Infrared Microwave Couple Product System) algorithm^77^. AIRS is a grating spectrometer aboard the second Earth Observing System (EOS) polar-orbiting platform, EOS Aqua, which was launched on May 4, 2002, into a polar sun-synchronous orbit. The CLIMCAPS algorithm uses an Optimal Estimation methodology with a-priori first guesses to start the process. SNDRAQIL3CMCCP product is a monthly product with global coverage of XCO_2_, with a spatial resolution of 1° and temporal coverage from September 2002 to July 2023. The data quality criteria are as follows: (a) Areas where the air pressure is less than 750 mB (areas of high altitude) and below 60 degrees south are marked in the visualization as low-quality data (striped areas); (b) Areas with data gaps and of high altitude less than 5% of the resolution of the product have been filled using the nearest neighbor algorithm.

The Global Land 1° Mapping-XCO_2_ dataset is available at 10.7910/DVN/4WDTD8^78^. The Mapping-XCO_2_ dataset is generated by the XCO_2_ retrievals from the Greenhouse Gases Observations Satellite (GOSAT) and Orbiting Carbon Observatory (OCO-2). This Mapping-XCO_2_ dataset is compiled on a monthly basis, with a spatial resolution of 1° and geographic coverage ranging from 56°S to 65°N and 169°W to 180°E. The uncertainties in this dataset are generally less than 1.5 parts per million (ppm). The spatiotemporal characteristics of global XCO_2_ in Mapping-XCO_2_ dataset are similar to those of the modeling data obtained from CarbonTracker^78^. Since the Mapping-XCO_2_ dataset is available from 2009 to 2020, we only used it to conduct spatial partial correlation analysis.

### Future climate data

To extend our findings for assessing future changes, we estimated phenology and greenness in 2050 using the projected monthly air temperature at all ozone sites from the Community Earth System Model 2 (CESM2) in the sixth phase of the Coupled Model Intercomparison Project (CMIP6) model simulations under SSP 5–8.5. This data has gone through a quality control procedure and ensures a high standard of dependability of the data (https://cds.climate.copernicus.eu/cdsapp#!/dataset/projections-cmip6?tab=form)^79^. The CMIP6 data underpins the Intergovernmental Panel on Climate Change 6th Assessment Report, providing estimates of future climate change and related uncertainties^80^. We selected the SSP5–8.5 scenario as a high-end stress test, representing a fossil fuel–intensive pathway that produces the greatest radiative forcing and extreme climate outcomes. This scenario provides a critical upper bound for assessing potential impacts and underscores the urgency of aggressive mitigation and adaptation efforts. We extracted 2-m temperature, surface precipitation, and surface RH and calculated VPD using RH and temperature at all ozone sites.

For future CO_2_, we used CO_2_ prediction data from the XCO_2_ dataset obtained by Cheng et al. ^81^ (10.5281/zenodo.5021361)^81^. This dataset provides global distributions of CO_2_ concentrations in the future under eight SSP and the representative concentration pathways scenarios from CMIP6, including SSP1–1.9, SSP1–2.6, SSP2–4.5, SSP3–7.0, SSP4–3.4, SSP4–6.0, SSP5–3.4, and SSP5–8.5. We selected the SSP5–8.5 scenario from XCO_2_ dataset for 2022–2050 to ensure consistency with the selected future temperature projection.

### Future ozone data

We used the simulated surface ozone from the Community Earth System Model version 2.1.3 (CESM2.1.3), employing its atmospheric component CAM4-chem, which incorporates full tropospheric O_3_-NO_x_-CO-VOC-aerosol chemistry based on the MOZART-4 mechanism^78^. The model was run in time-slice mode at ~1° horizontal resolution (0.9° latitude × 1.25° longitude) with 26 vertical levels extending to ~40 km. Land–atmosphere processes were represented by the Community Land Model version 5 (CLM5) with active biogeochemistry, in which biogenic VOC emissions were calculated online using MEGANv2.1 as a function of vegetation type, meteorology, and CO_2_ levels, while dry deposition was dynamically linked to stomatal conductance. Anthropogenic and biomass burning emissions followed the CMIP6 inventory, while future scenarios of land use, greenhouse gases, and short-lived species were prescribed from SSP. Simulations included factorial experiments isolating the effects of land-use change, climate change, and emissions, as well as combined forcings, each integrated for 15 years with the first 5 years discarded for spin-up.

### Calculation of AOT40

In this study, we used AOT40 to evaluate the influence of ozone on vegetation phenology and greenness. The AOT40 (Accumulated Ozone Exposure Over a Threshold of 40 parts per billion volume, ppbv) is a standard metric in atmospheric science and environmental monitoring to assess the phytotoxic effects of elevated ground-level ozone concentrations on vegetation^82,83^. Numerous studies have established 40 ppbv as the critical threshold for ozone-induced vegetation damage, making AOT40 a widely adopted measure for quantifying cumulative ozone exposure above this threshold^84^.

Unlike traditional methods that calculate AOT40 based only on daytime measurements (8 AM to 8 PM), we included a full 24-h period to provide a more accurate representation of ozone exposure. This approach captures potential vegetation impacts occurring both day and night, offering deeper insight into ozone’s influence on plant growth cycles. The formula used for this calculation is as follows:

4 $${AOT}40={\sum }_{8}^{20}\max \left([C{{{\rm{ozone}}}}-40],0\right)$$

where C_ozone_ is the hourly ozone concentration (ppbv), and AOT40 is daily value.

We calculated AOT40 values for different seasonal periods from daily value: spring (March 1st to May 31st), autumn (September 1st to November 31st), summer (June 1st to August 31st), and all-year (January 1st to December 31st), respectively. These seasonal and annual AOT40 were then used in the partial correlation analysis with key phenological metrics, including the SOS, EOS, EVI_max_, and EVI_area_.

### Partial correlation analysis

In this study, we used the partial correlation method to investigate the relationship between ozone pollution and vegetation indices by eliminating the influence of other environmental factors. The partial correlation between *X* (AOT40, or surface ozone level) and *Y* (e.g., SOS, EOS, EVI_max_, or EVI_max_), is denoted as $R\left(X,Y,|,Z\right)$, where Z represents conditions on sets of environmental covariates to be excluded in the analysis. The partial correlation coefficient is calculated as the residual correlation between *X* and *Y* after removing the linear influence of *Z* as follows:

5 $$R\left(X,Y,|,Z\right)=\frac{R\left(X,Y\right)-R\left(X,Z\right)\cdot R\left(Y,Z\right)}{\sqrt{\left(1-{R}^{2}\left(X,Z\right)\right)\left(1-{R}^{2}\left(Y,Z\right)\right)}}$$

We performed partial correlation analyses at two scales: spatial and temporal. Spatial partial correlation analysis conducted using data from all sites within a single year and repeated annually, representing the spatial relationship of environmental factors on vegetation. The temporal analysis was conducted for each site using the time series data spanning all years, thereby examining the temporal relationship of environmental factors with vegetation at each site. We obtained the spatial and temporal partial correlation coefficients between environmental variables, including AOT40, temperature, precipitation, VPD, and CO_2_ concentrations and vegetation metrics (SOS, EOS, EVI_max_, and EVI_max_) (e.g., R_AOT40-SOS_, R_AOT40-EOS_, R_AOT40-EVImax_, and R_AOT40-EVIarea_). Previous studies have reported that changes in CO_2_ significantly impact vegetation greenness but not much on vegetation phenology^85,86^. Therefore, we included CO_2_ in our analysis of vegetation greenness (EVI_max_ and EVI_area_), but not in vegetation phenology analysis (SOS or EOS)^7,87,88^.

### Machine learning model

To assess the potential impacts of future climate and ozone changes on vegetation, we developed a machine-learning model (LightGBM), trained on historical vegetation and environmental data. LightGBM is a highly efficient gradient boosting framework and powerful machine learning technique that demonstrates remarkable capabilities in handling complex nonlinear relationships and mitigating the adverse effects of multicollinearity in high-dimensional datasets. The key innovation of LightGBM model is based on the leaf-wise tree growth strategy, which prioritizes optimal splits to better capture nonlinear patterns compared to traditional depth-wise methods. The LightGBM model incorporates two advanced techniques: Gradient-based One-Side Sampling for instance selection and Exclusive Feature Bundling for dimensionality reduction.

In the LightGBM model, the dependent variable (Y) can be expressed as a complex function of multiple independent variables (X_1_, X_2_, X_3_, X_4_, X_5_) as follows:

6 $$y=f({X}_{1},{X}_{2},{X}_{3},{X}_{4},{X}_{5})$$

where *y* is the observed vegetation phenology and productivity data. The multiple independent variables (X_1_, X_2_, X_3_, X_4_, X_5_) are AOT40, temperature, precipitation, and VPD for SOS, EOS, and the AOT40, temperature, precipitation, VPD, and CO_2_ for EVI_max_ and EVI_area_. The metrics used for assessing the performance of the MLR models include the Pearson Correlation (R), Root-Mean-Square Error (RMSE), and Mean Absolute Error

We also utilized SHAP to interpret the predictions of the LightGBM model. SHAP is an explainable AI analysis that quantifies the contribution of individual features to model outputs. SHAP analysis revealed the relative importance of key variables (e.g., AOT40, temperature, and CO₂) in driving predictions of vegetation phenology and productivity. Specifically, positive (negative) SHAP values indicate features that increase (decrease) the predicted outcome relative to the baseline, which is a positive correlation (negative correlation).

### Future predictions

We conducted two simulations using machine learning model LightGBM to quantify ozone’s effect in vegetation changes (SOS, EOS, EVI_max_, and EVI_area_) for all sites over the USA, Europe, and China in 2050. First, we held ozone concentrations fixed at 2022 level while using temperature, precipitation, VPD, and CO_2_ projections from the CMIP6 under SSP5–8.5. Second, we incorporated both climate variables from CMIP6 and the ozone projections from Bhattarai et al. ^89^ under SSP5–8.5. The difference between these two predictions specifically isolates ozone’s contribution to future vegetation changes.

For each site, we calculated the spring (March 1st to May 31st), autumn (September 1st to November 31st), summer (June 1st to August 31st), and all-year (January 1st to December 31st) mean temperature, precipitation, relative humidity, and XCO_2_ extracted from CMIP6 model in 2050. We used temperature and RH to calculate VPD. Then, we used spring AOT40, temperature, precipitation, and VPD to predict SOS; autumn AOT40, temperature, precipitation, and VPD to predict EOS; summer AOT40, temperature, precipitation, and VPD to predict EVI_max_; and all-year AOT40, temperature, precipitation, and VPD to predict EVI_area_.

Our LightGBM model demonstrated strong performance in reproducing historical trends and variability across all study sites (Fig. S23), validating its application for future projections. Meanwhile, we compared the distributions of predictor variables between the training dataset and future scenarios. Meteorological variables, including VPD, temperature, and precipitation, remained within the range of the training data, indicating limited extrapolation for climate drivers, while increases in ozone and CO_2_ reflect the prescribed scenario assumptions (Fig. S26). Using this framework, we estimated changes in vegetation phenology and greenness under future conditions for 2050 across the USA, Europe, and China with and without considering ozone effect.

### Reporting summary

Further information on research design is available in the Nature Portfolio Reporting Summary linked to this article.

## Supplementary information

Supplementary Information

Peer Review file

Reporting Summary

## Data Availability

The ground-based ozone data are obtained from the China National Environmental Monitoring Centre (CNEMC) network (http://www.cnemc.cn/en/), the European Environment Agency (EEA, https://www.eea.europa.eu/), and the United States Environmental Protection Agency (EPA, https://www.epa.gov/outdoor-air-quality-data/download-daily-data). The land cover type data is extracted from MODIS Land Cover Type (MCD12Q1) Version 6.1 data product (https://lpdaac.usgs.gov/products/mcd12q1v061/). The vegetation phenology and greenness datasets are obtained from NASA Visible Infrared Imaging Radiometer Suite (VIIRS) Global Land Surface Phenology (GLSP) data product (https://lpdaac.usgs.gov/products/vnp22q2v001/). The meteorological dataset are extracted from ERA5-Land climate reanalysis hourly dataset from the European Center for Medium-Term Weather Forecasting (ECMWF, https://climate.copernicus.eu/climate-reanalysis). The historical XCO_2_ datasets are extracted from SNDRAQIL3CMCCP and Global land 1° Mapping-XCO_2_ [10.7910/DVN/4WDTD8]. The future predictions datasets are extracted from the Community Earth System Model 2 (CESM2) in the sixth phase of the Coupled Model Intercomparison Project (CMIP6) model simulations under Shared Socioeconomic Pathways (SSP) 5–8.5 [https://cds.climate.copernicus.eu/cdsapp#!/dataset/projections-cmip6?tab=form]. CO_2_ prediction data is from the XCO_2_ dataset obtained by Cheng et al. ^81^ [10.5281/zenodo.5021361].

## Code availability

All data analysis and figures for this study were performed or created using Python v3.11. The code is available at: [10.5281/zenodo.18826646]^90^. Code reuse is encouraged.
