# Supplementary material for: Ozone mitigates extended growing season and enhanced vegetation greenness driven by environmental change
Source: Nat Commun. 2026 Apr 20;17:5445. doi: 10.1038/s41467-026-71959-y (PMC13280477; doi:10.1038/s41467-026-71959-y)
Supplement: Supplementary file 1 — Supplementary Information [file 41467_2026_71959_MOESM1_ESM.pdf]

Supplementary Materials

Ozone mitigates extended growing season and enhanced vegetation greenness driven by environmental change

Hao Yin[1], Lin Meng[1, *], Andrew D. Richardson[2,3], Maria Val Martin[4], Jiafu Mao[5], Huidong Li[1], Jonathan M. Gilligan[1], Hemraj Bhattarai[6], Amos P. K. Tai[7,8]

[1] Department of Earth and Environmental Science, Vanderbilt University, Nashville, TN, USA

[2] Center for Ecosystem Science and Society, Northern Arizona University, Flagstaff, AZ, USA

[3] School of Informatics, Computing, and Cyber Systems, Northern Arizona University, Flagstaff, AZ, USA

[4] Leverhulme Centre for Climate Change Mitigation, School of Biosciences, University of Sheffield, Sheffield, S10 2TN, UK

[5] Environmental Sciences Division, Oak Ridge National Laboratory, Oak Ridge, TN, USA

[6] Division of Environment and Sustainability, The Hong Kong University of Science and Technology, Hong Kong, China

[7] Department of Earth and Environmental Sciences, The Chinese University of Hong Kong, Hong Kong, China

[8] State Key Laboratory of Agrobiotechnology, and Institute of Environment, Energy and Sustainability, The Chinese University of Hong Kong, Hong Kong, China

[*] Corresponding Author: Lin Meng (lin.meng@vanderbilt.edu)

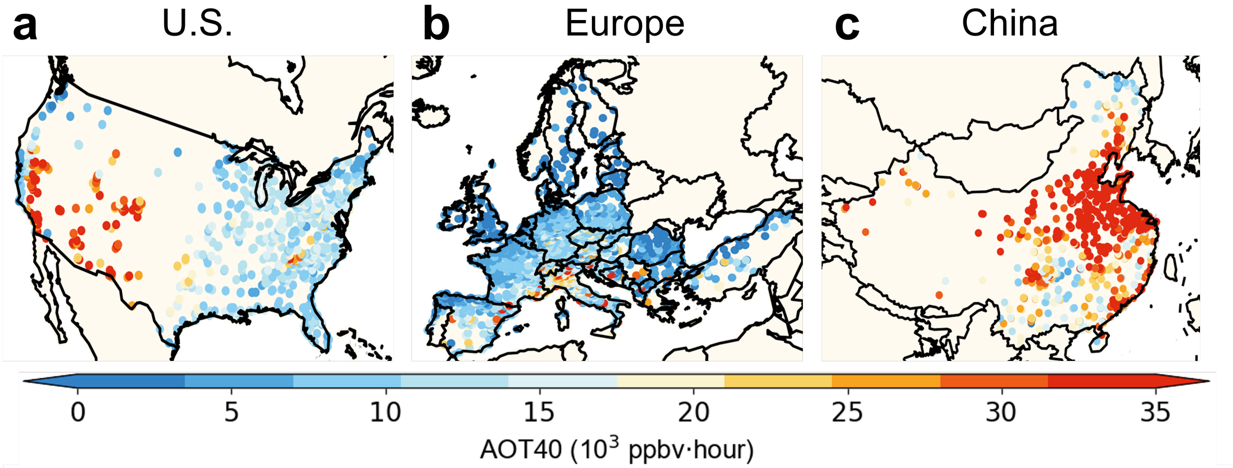

Figure S1. Spatial patterns of long-term averaged Accumulated dose of ozone Over a Threshold of 40 ppbv (AOT40) over the U.S. (**a**), Europe (**b**), and China (**c**). Study period is 2013-2022 for U.S. and Europe, and 2015-2022 for China.

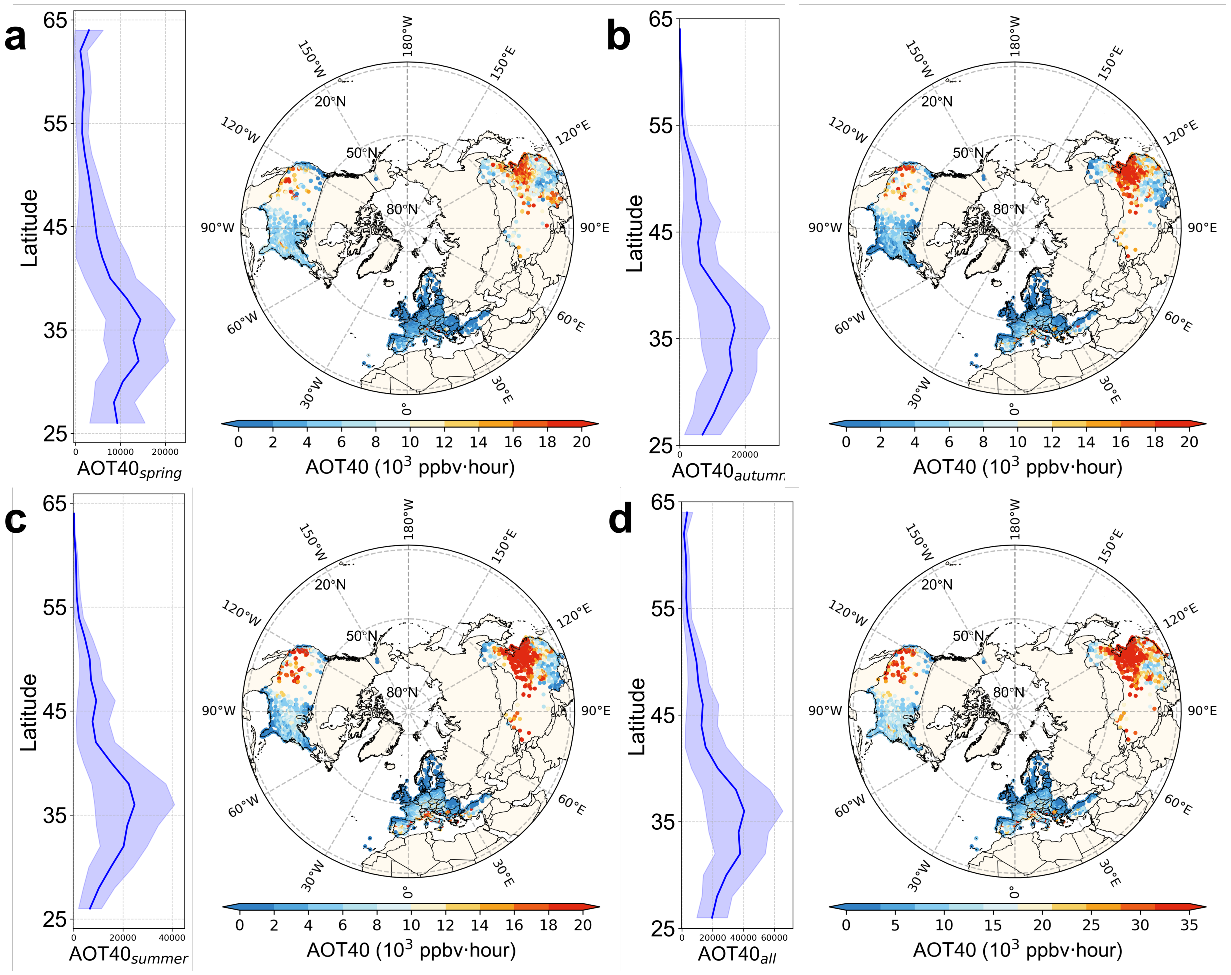

Figure S2. The spatial patterns of averaged AOT40 in spring (**a**), autumn (**b**), summer (**c**) and all year (**d**), with the variation of each variable with latitude.

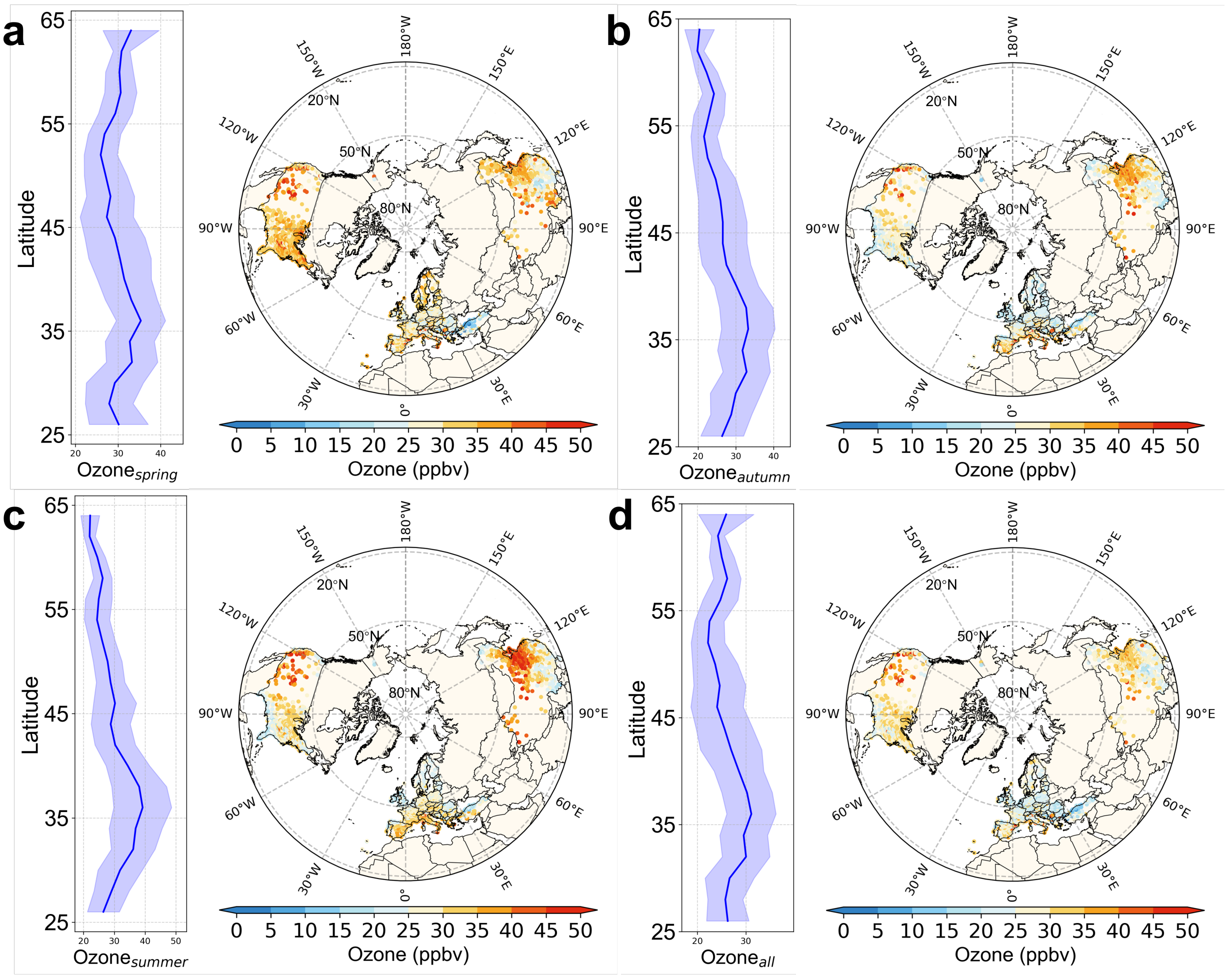

Figure S3. The spatial patterns of averaged ozone levels in spring (**a**), autumn (**b**), summer (**c**), and all year (**d**), with the variation of each variable with latitude.

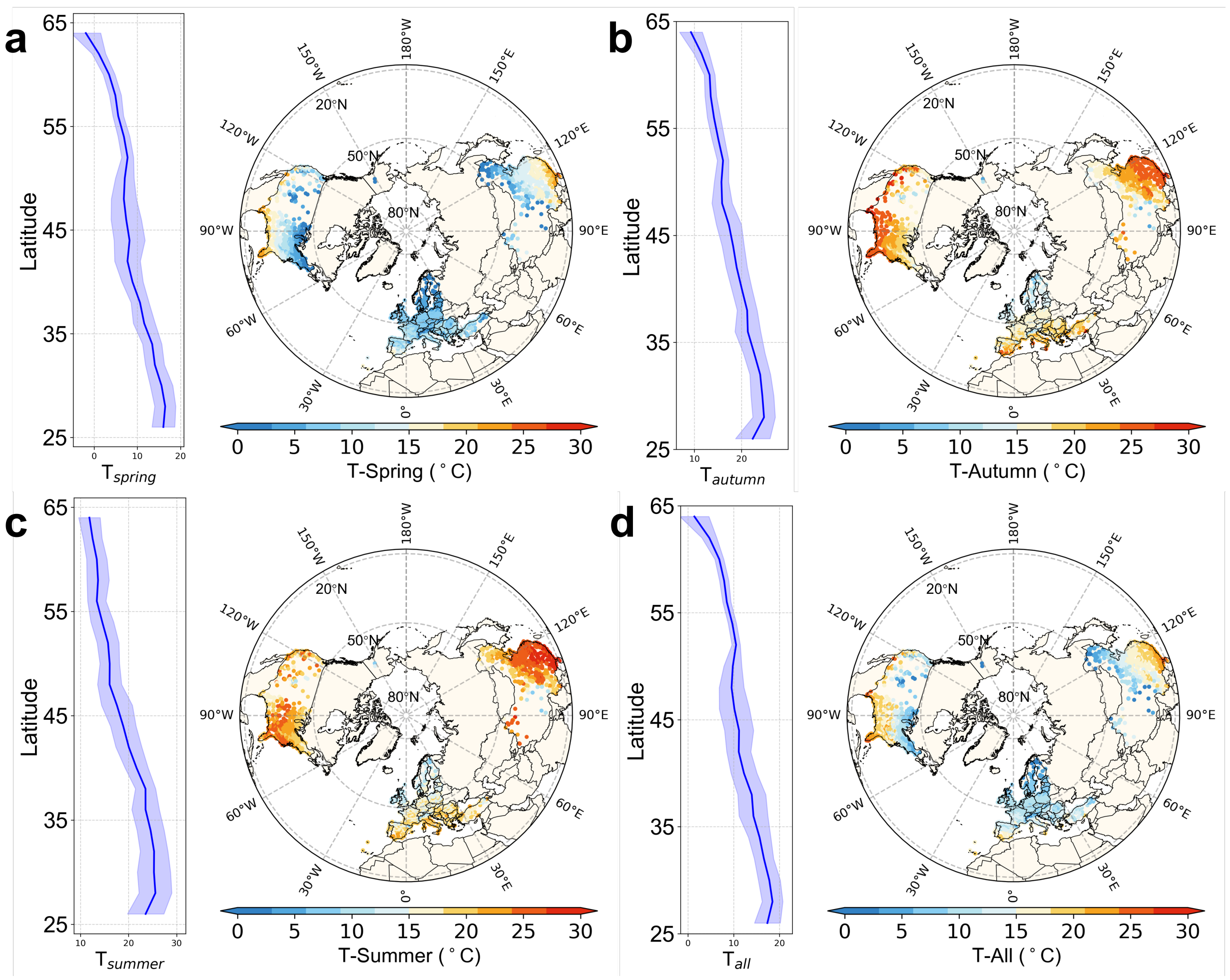

Figure S4. The spatial patterns of averaged temperature in spring (**a**), autumn (**b**), summer (**c**), and all year (**d**), with the variation of each variable with latitude.

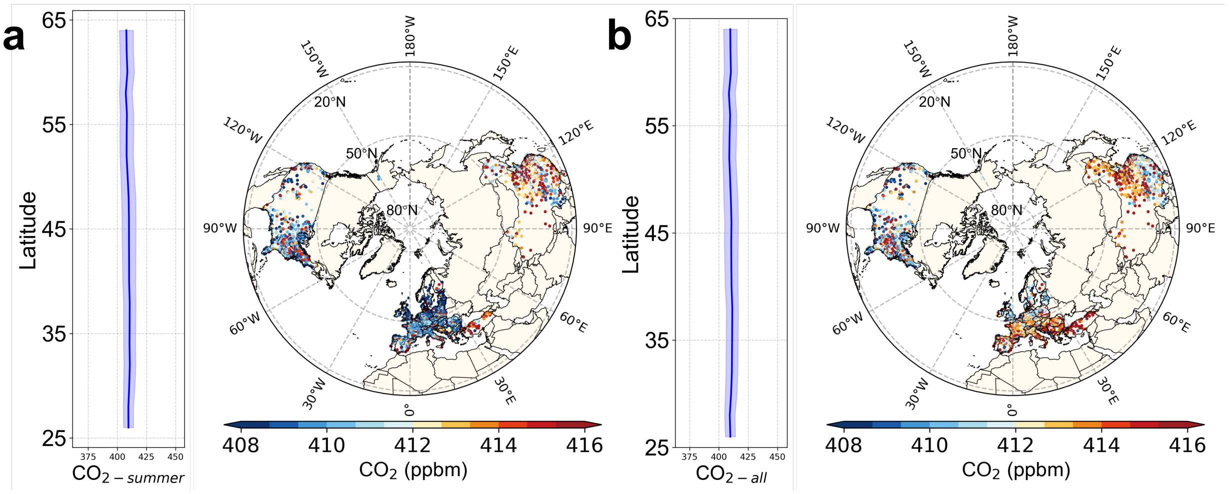

Figure S5. The spatial patterns of averaged XCO_2 in summer (**a**), and all year (**b**) based on XCO_2 dataset, with the variation of each variable with latitude.

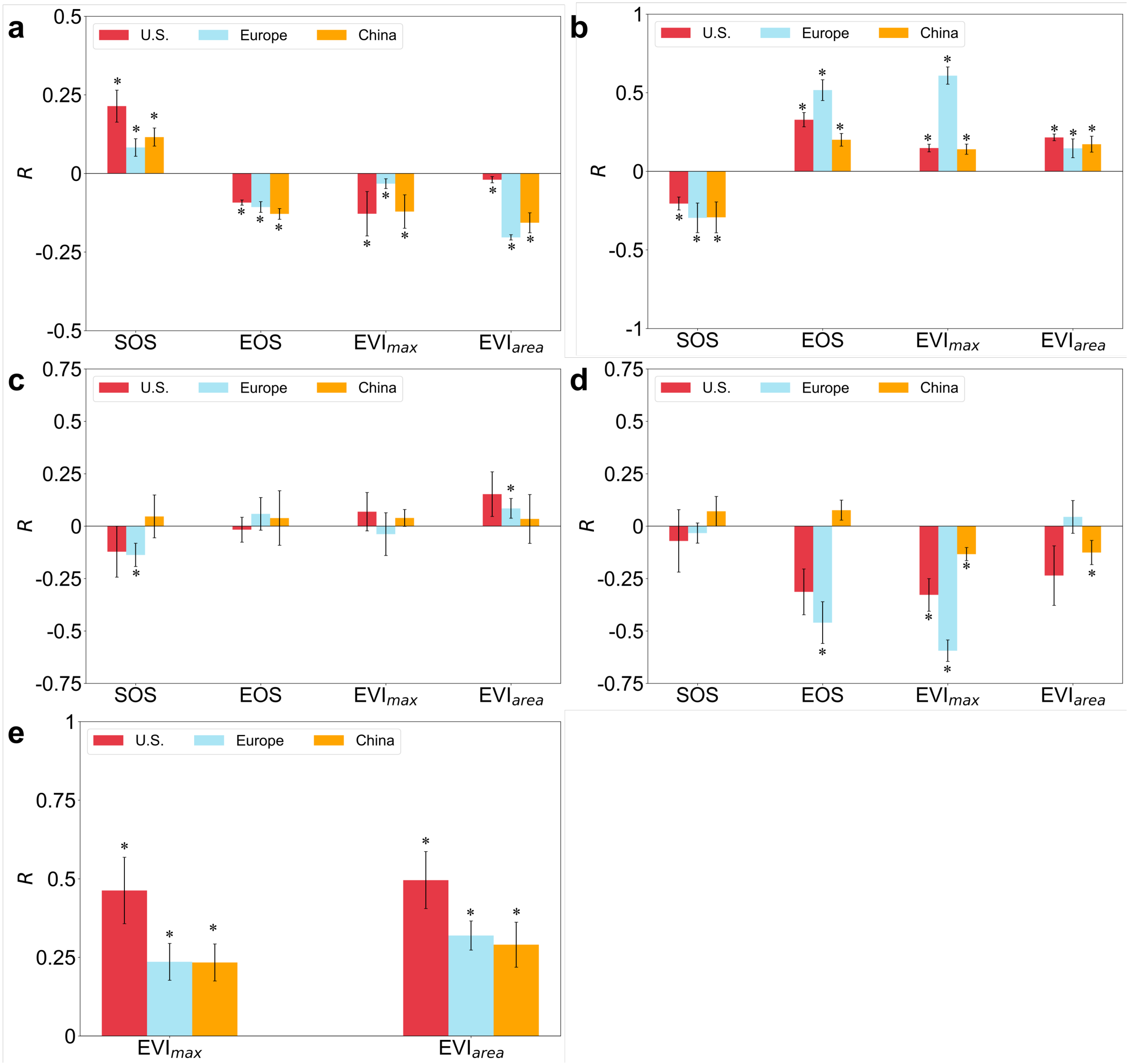

Figure S6. Partial correlation coefficients (*R*) between ozone concentrations (**a**); temperature (**b**); VPD (**c**); Precipitation (**d**); XCO_2 (**e**); and vegetation indexes for U.S., Europe, and China. The bar represents mean values and error bars represent the standard deviations of partial correlation coefficients across years. Asterisk denotes statistically significant at the 95% confidence level ($p < 0.05$) using two sided tests.

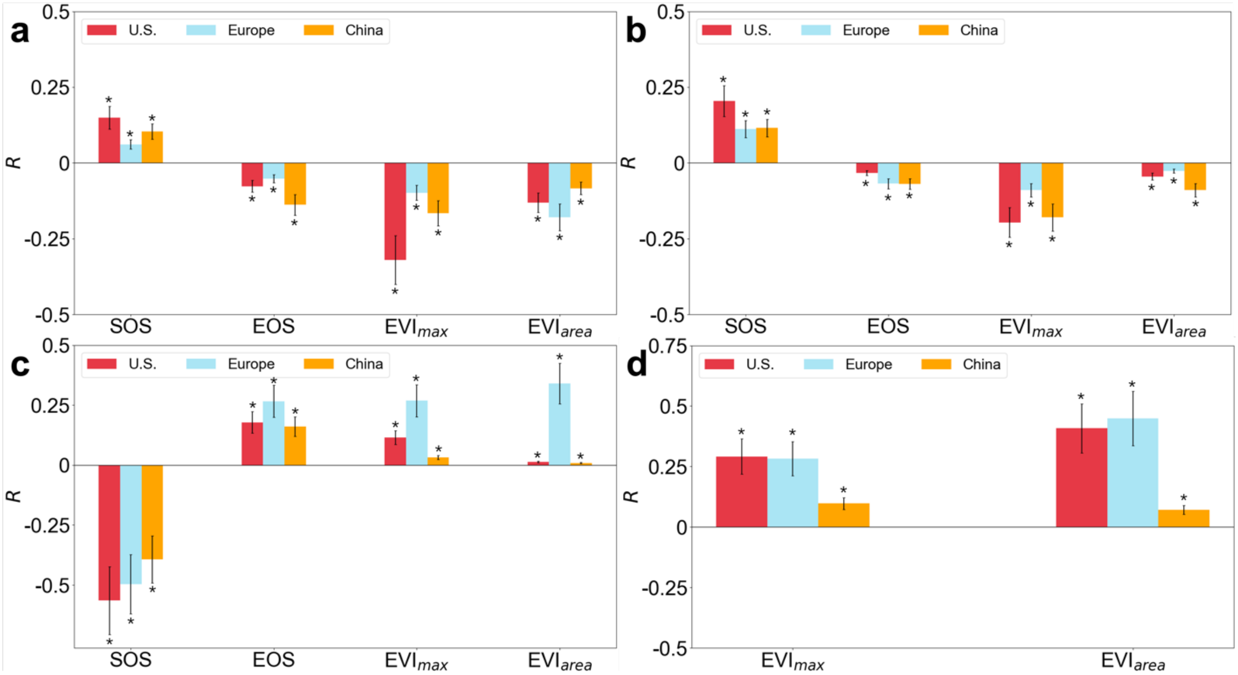

Figure S7. Partial correlation coefficients (*R*) between vegetation indexes and AOT40 (**a**), ozone concentrations (**b**), temperature (**c**), XCO_2 (**d**) for U.S., Europe, and China. For (a) and (b), temperature, XCO_2, precipitation, and VPD are control variables. The bar represents mean values and error bars represent the standard deviations of partial correlation coefficients across years. Asterisk denotes statistically significant at the 95% confidence level ($p < 0.05$) using two sided tests. XCO_2 was obtained from GOSAT Global land 1° mapping dataset.

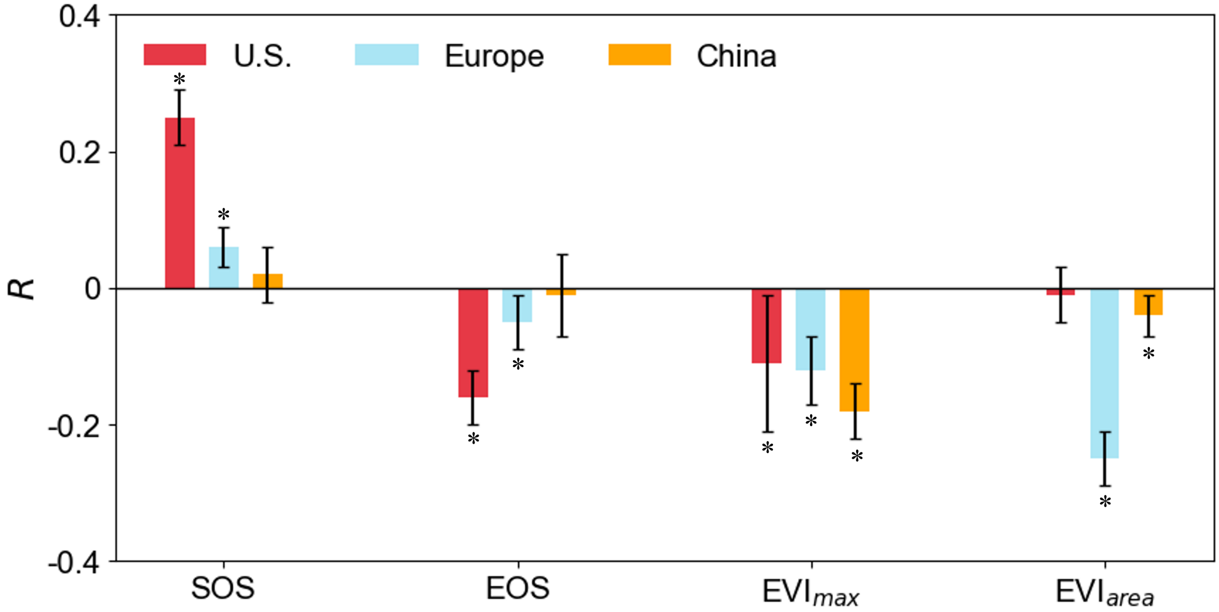

Figure S8. Partial correlation coefficients (*R*) between AOT40 and SOS, EOS, EVI_{max} and EVI_{area}., respectively, for U.S., Europe, and China. Same with Figure 2e, but using MODIS satellite data. The bar represents mean values and error bars represent the standard deviations of *R* across years. The asterisk denotes statistical significance at the 95% confidence level ($p < 0.05$) using two sided tests.

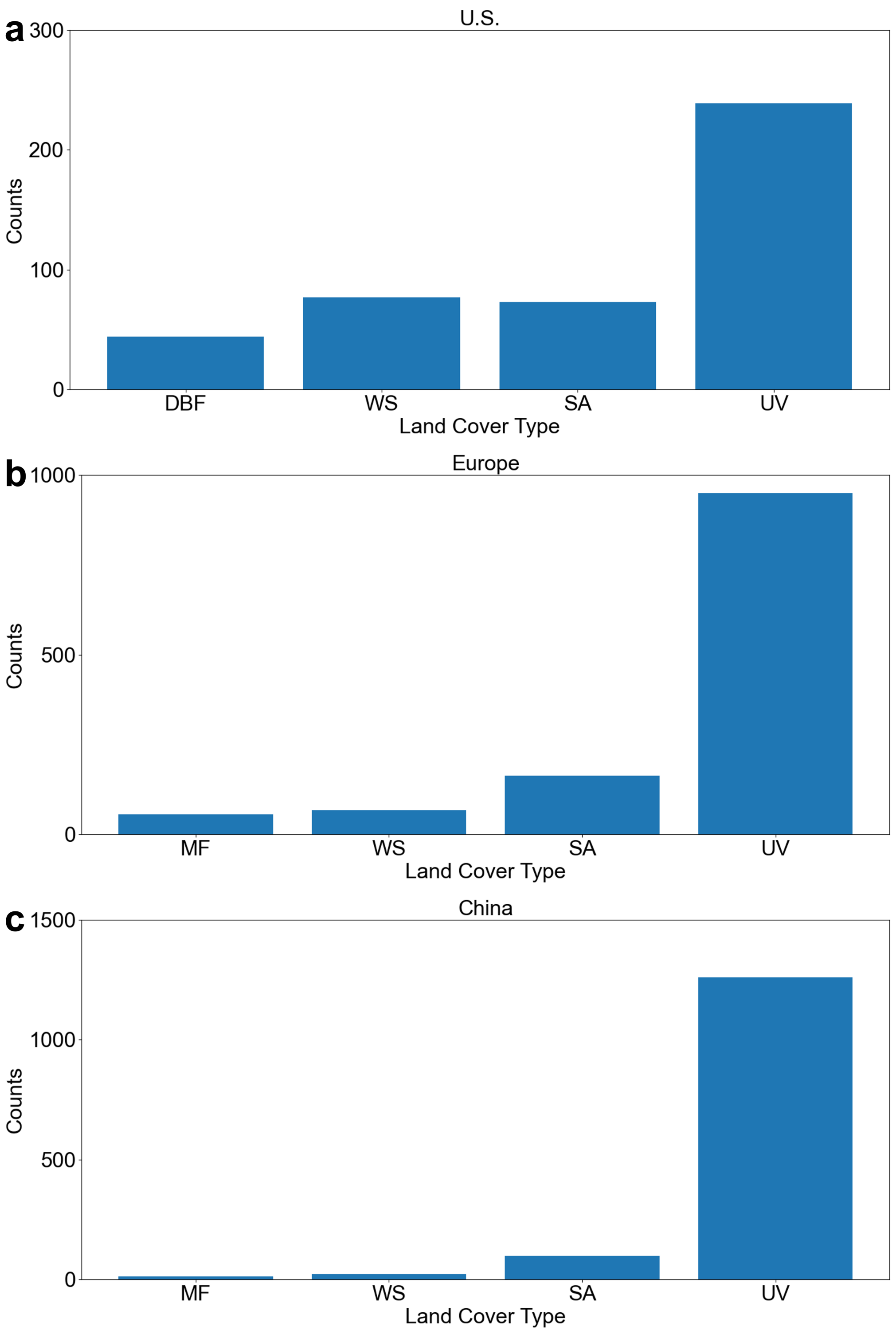

Figure S9. The land cover types for study sites in U.S. (**a**), Europe (**b**), and China (**c**). The DBF, MF, WS, SA, UV are Deciduous Needleleaf Forests, Mixed Forests, Woody Savannas, Savannas, Urban Vegetation, respectively.

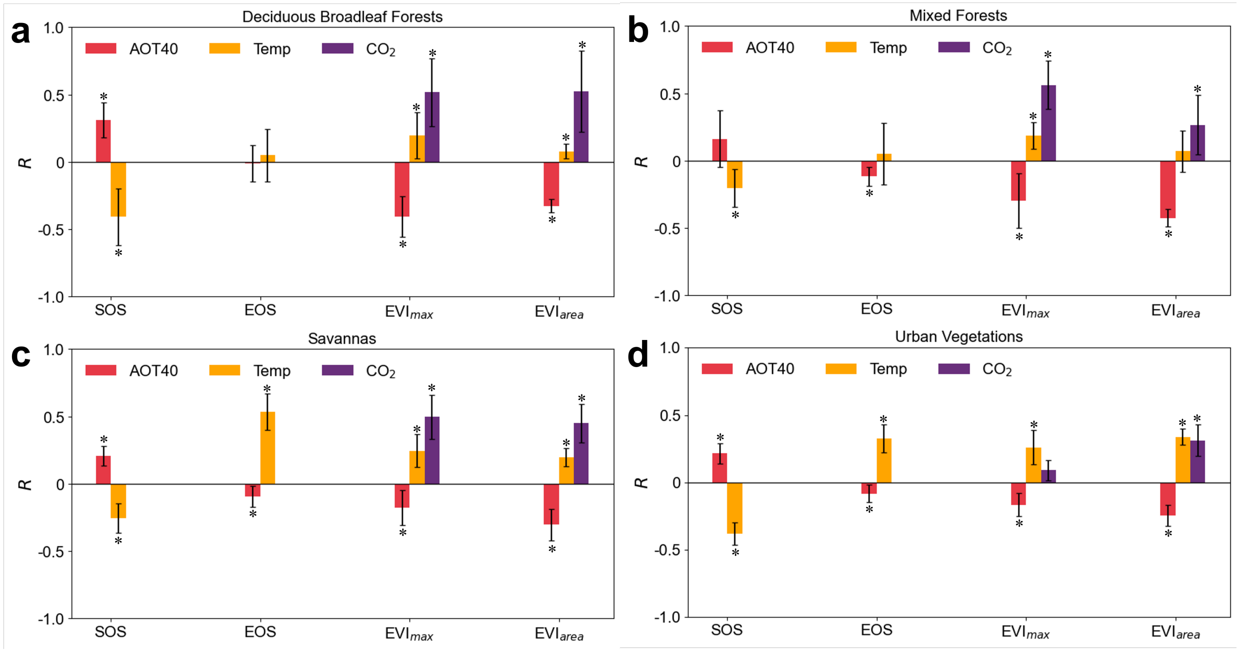

Figure S10. Same with Figure 2e, but for each land cover type (**a.** Deciduous Broadleaf Forests, **b.** Mixed Forests, **c.** Savannas, **d.** Urban Vegetations). The bar represents mean values and error bars represent the standard deviations of partial correlation coefficients across different years. Asterisk denotes statistically significant at the 95% confidence level ($p < 0.05$).

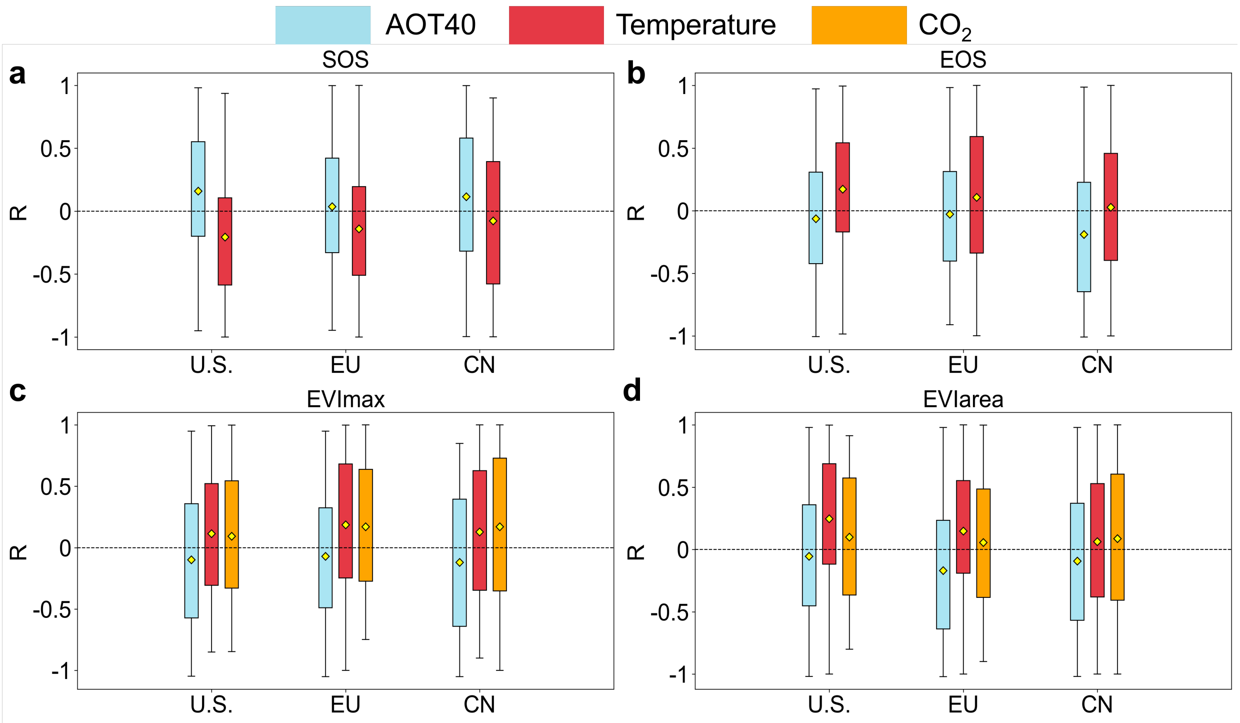

Figure S11. The effects of AOT40, temperature, and CO_2 on start of season (SOS) (**a**), end of season (EOS) (**b**), maximum EVI (EVI_{max}) (**c**), and cumulative EVI throughout the growing season (EVI_{area}) (**d**). Same as Figure 3e., but for each study region. The diamonds indicate the average of partial correlations; box edges denote 25% and 75% quartiles; the whiskers represent 25% of the standard deviation.

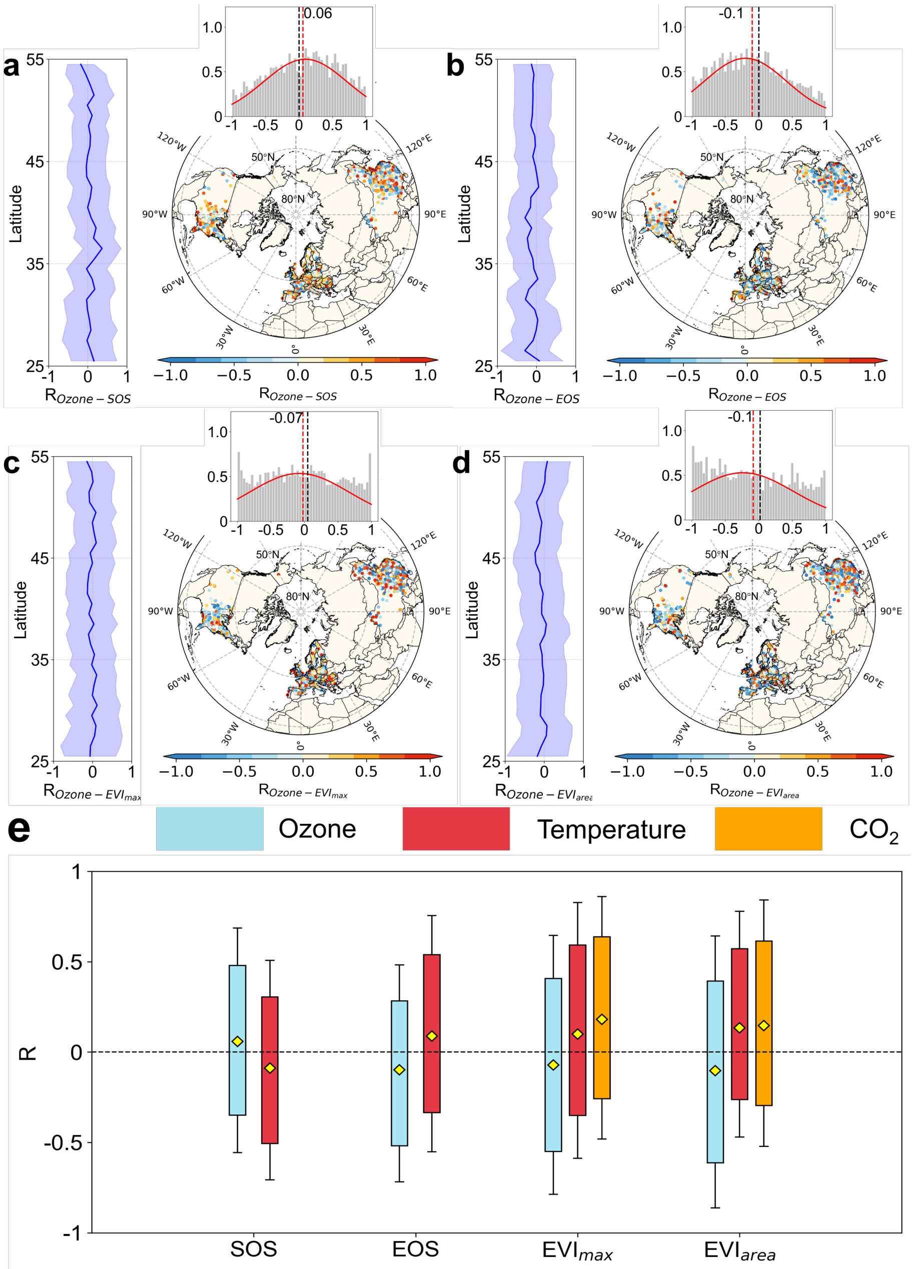

Figure S12. Same as Figure 3, but using ozone concentrations instead of AOT40. **a-d:** Spatial patterns of temporal partial correlation coefficients (*R*) between ozone concentrations and start of season (SOS) (**a**), end of season (EOS) (**b**), maximum EVI (EVI_{max}) (**c**), and cumulative EVI throughout the growing season (EVI_{area}) (**d**). The inserted subplots above the map show histograms of *R*. Black dashed lines indicate zero values, while red dashed lines and numbers represent the mean coefficients. Left subplots show the changes with latitude, with lines indicate the averaged R and shadow indicates the standard variances. **e:** Partial correlation coefficients between ozone concentrations, temperature, CO_2 and vegetation phenology and greenness (n=24426). The diamonds indicate the average of partial correlations; box edges denote 25% and 75% quartiles; the whiskers represent 25% of the standard deviation.

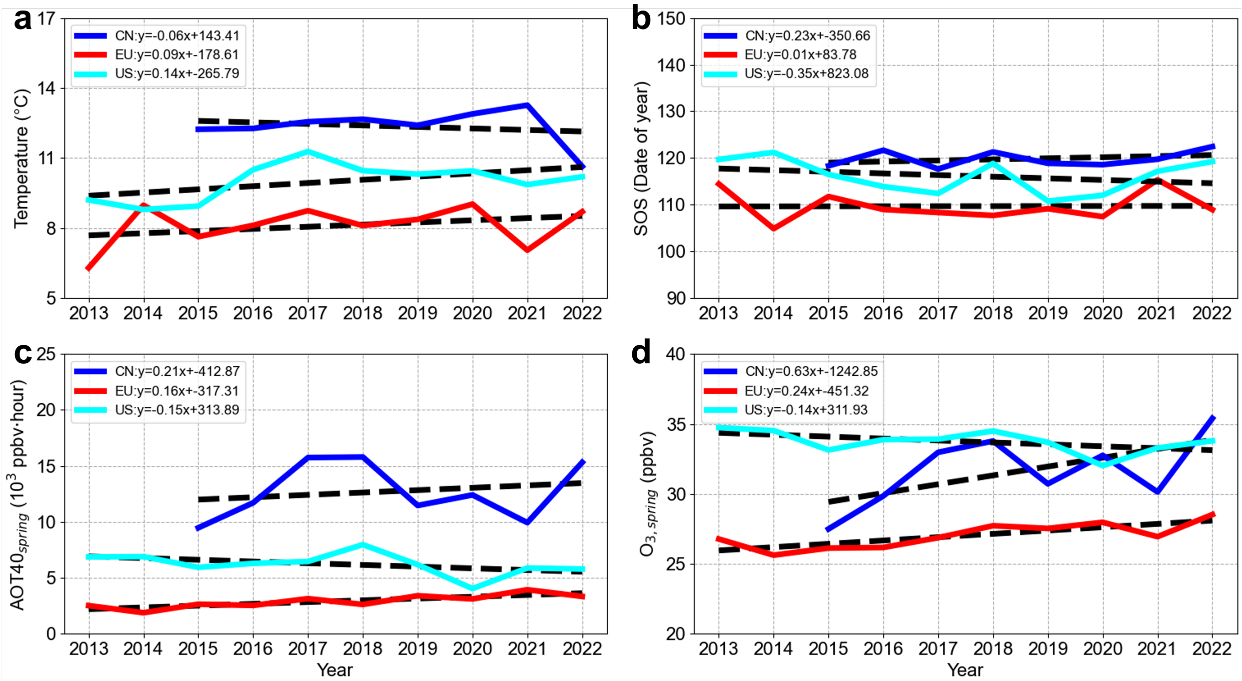

Figure S13. Time series of spring averaged temperature (**a**), SOS (**b**), spring AOT40 (**c**), and spring ozone concentrations (**d**) in U.S., Europe, and China. Black dashed lines represent fitted linear regressions in each region.

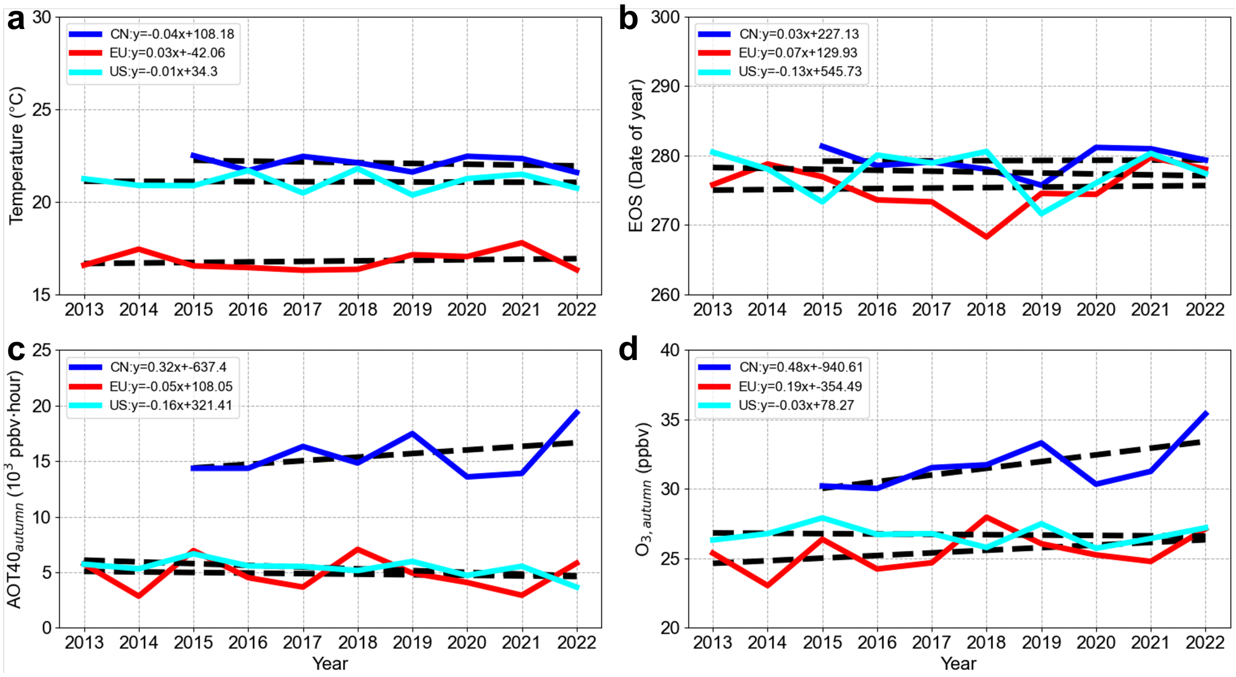

Figure S14. Time series of autumn averaged temperature (**a**), EOS (**b**), autumn AOT40 (**c**), and autumn ozone concentrations (**d**) in U.S., Europe, and China. Black dashed lines represent fitted regressions in each region.

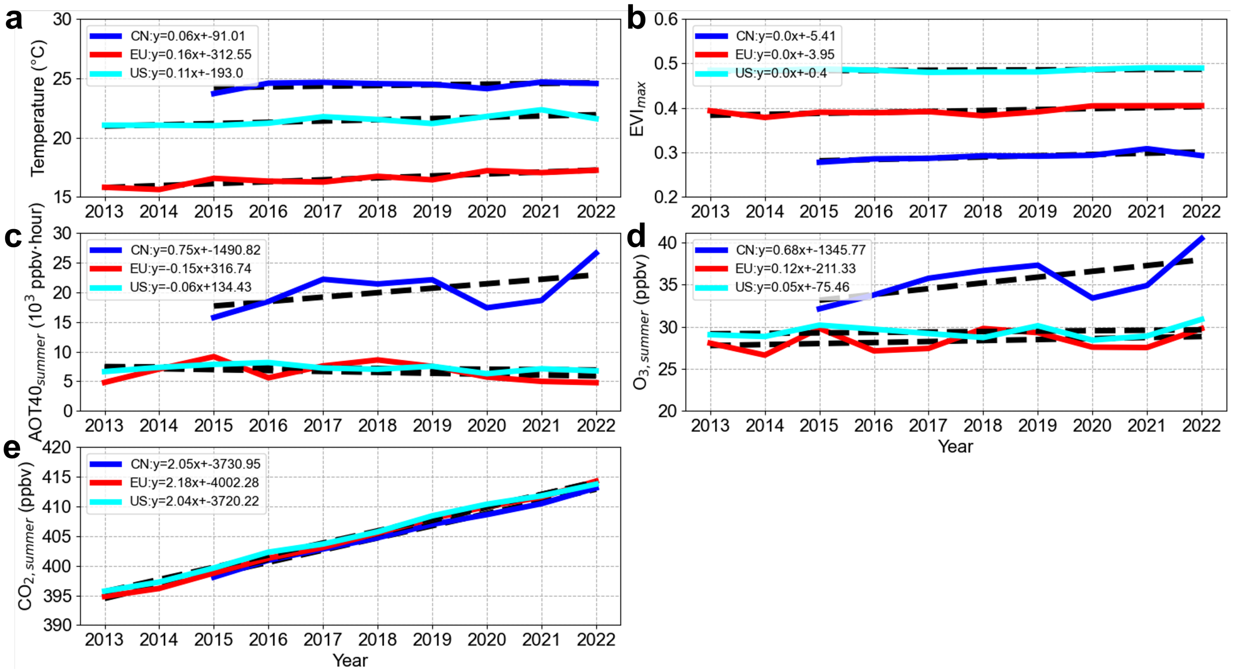

Figure S15. Time series of summer averaged temperature (**a**), EVI_{max} (**b**), summer AOT40 (**c**), summer ozone concentrations (**d**), and summer CO_2 concentrations (**e**) in U.S., Europe, and China. Black dashed lines represent fitted regressions in each region.

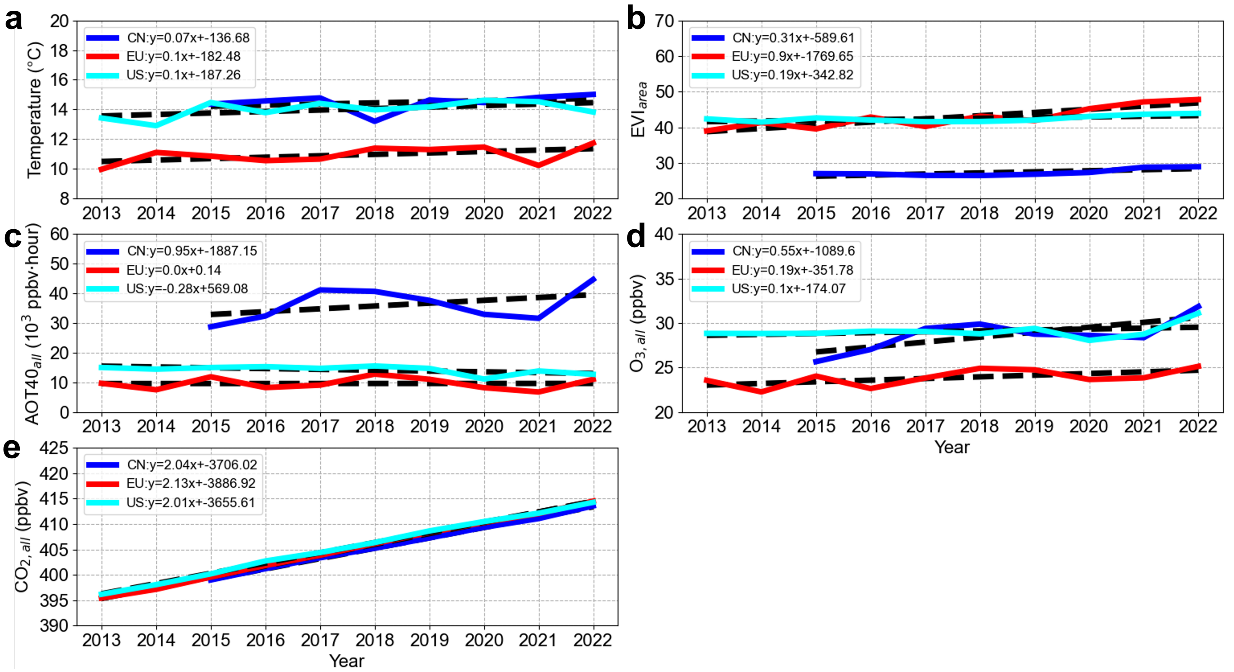

Figure S16. Time series of annual averaged temperature (**a**), EVI_{area} (**b**), annual averaged AOT40 (**c**), annual averaged ozone concentrations (**d**), and annual averaged CO_2 concentrations (**e**) in U.S., Europe, and China. Black dashed lines represent fitted regressions in each region.

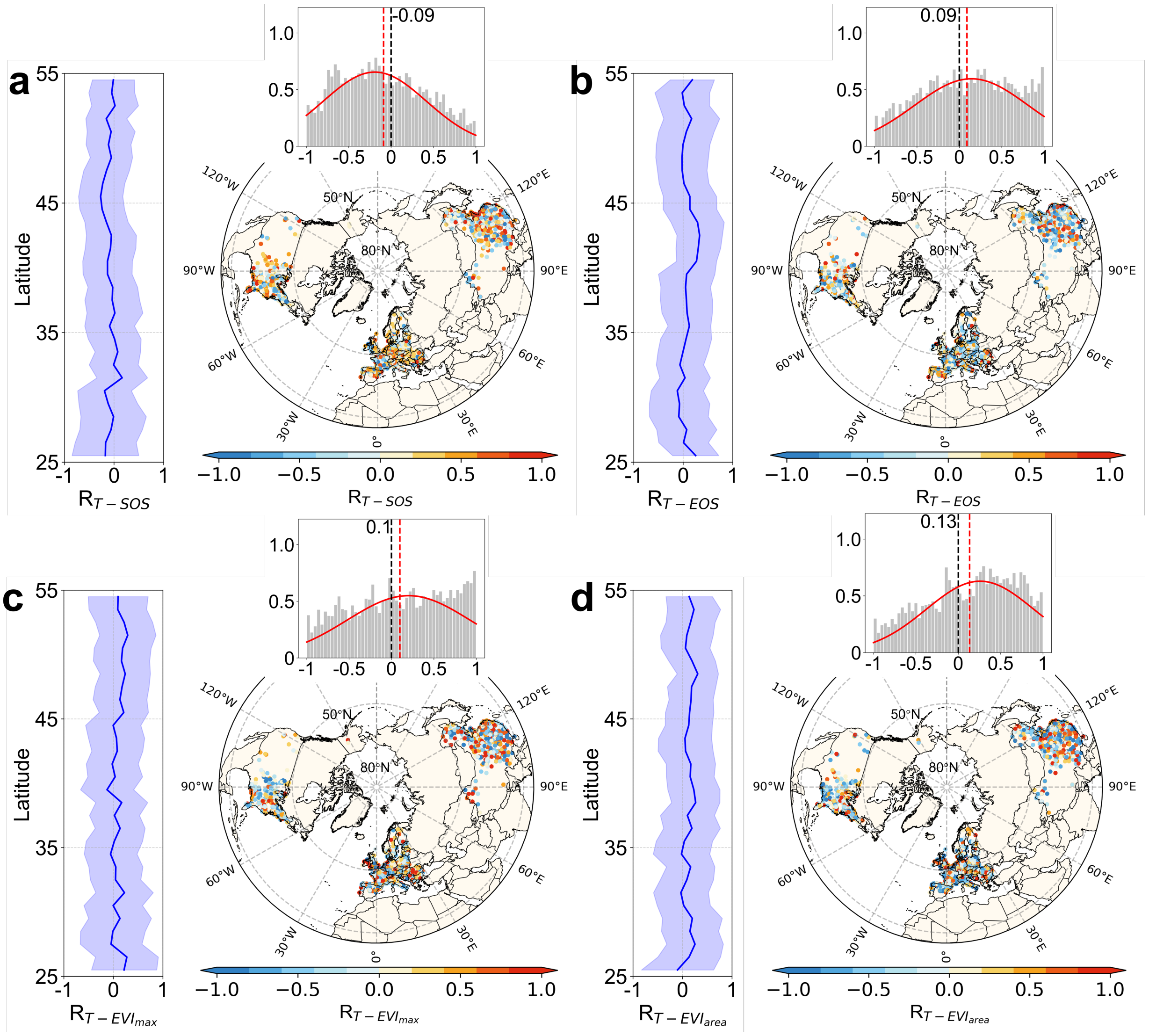

Figure S17. Same as Figure 3, but for partial correlation coefficients between temperature and start of season (SOS) (**a**), end of season (EOS) (**b**), maximum EVI (EVI_{max}) (**c**), and cumulative EVI throughout the growing season (EVI_{area}) (**d**).

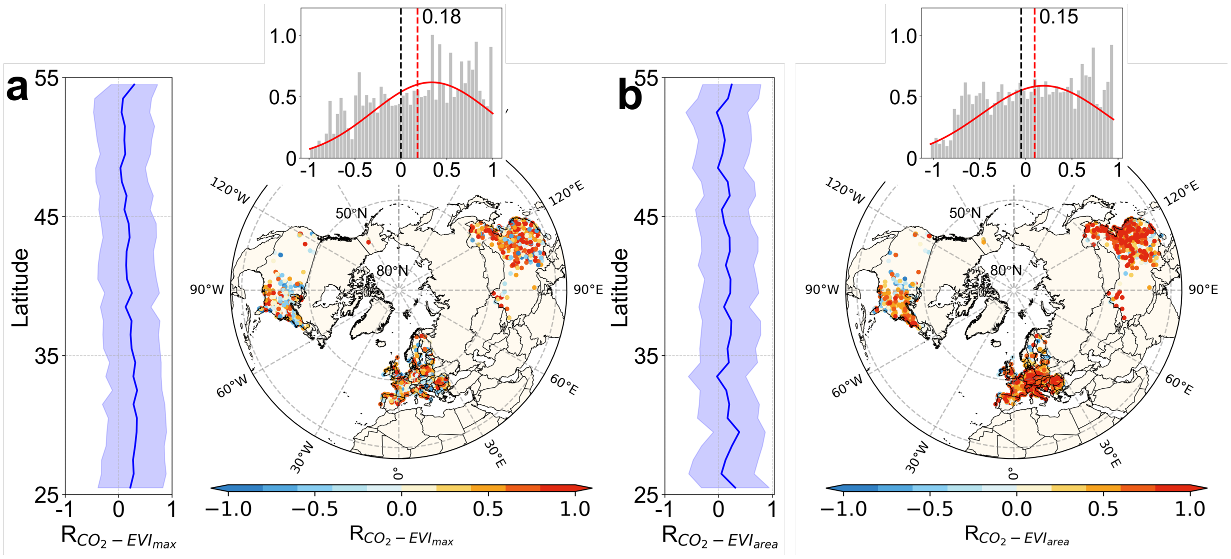

Figure S18. Same as Figure 3, but for partial correlation coefficients between XCO_2 and maximum EVI (EVI_{max}) (**a**) and cumulative EVI throughout the growing season (EVI_{area}) (**b**).

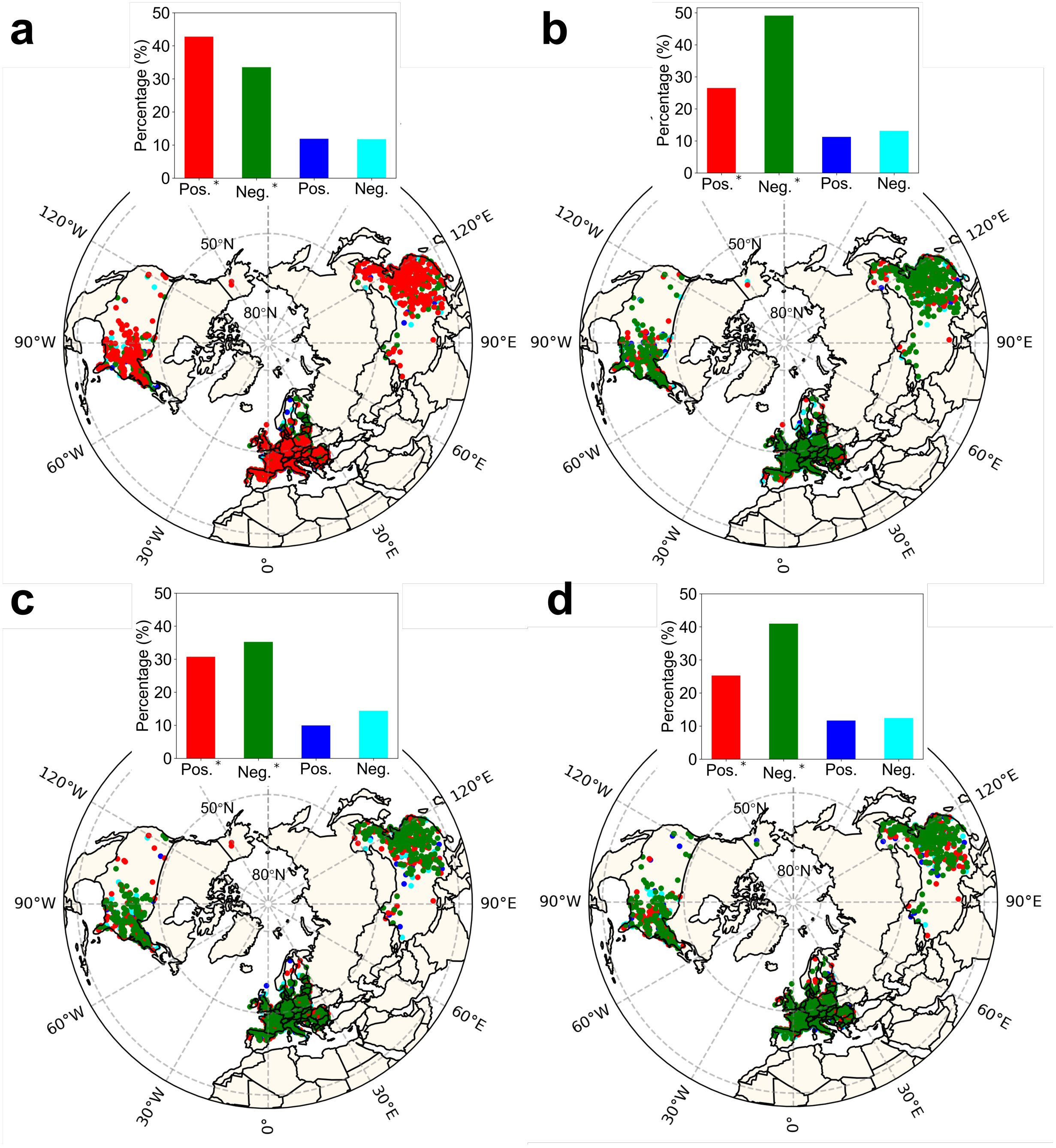

Figure S19. Spatial patterns of statistically significance at the 95% confidence level ($p < 0.05$) for partial correlation analysis between AOT40 and SOS (**a**), EOS (**b**), EVI_{max} (**c**) and EVI_{area} (**d**). Colors represent the significant positive correlations (Pos.*), significant negative correlations (Neg.*), non-significant positive correlation (Pos.), and non-significant negative correlation (Neg.).

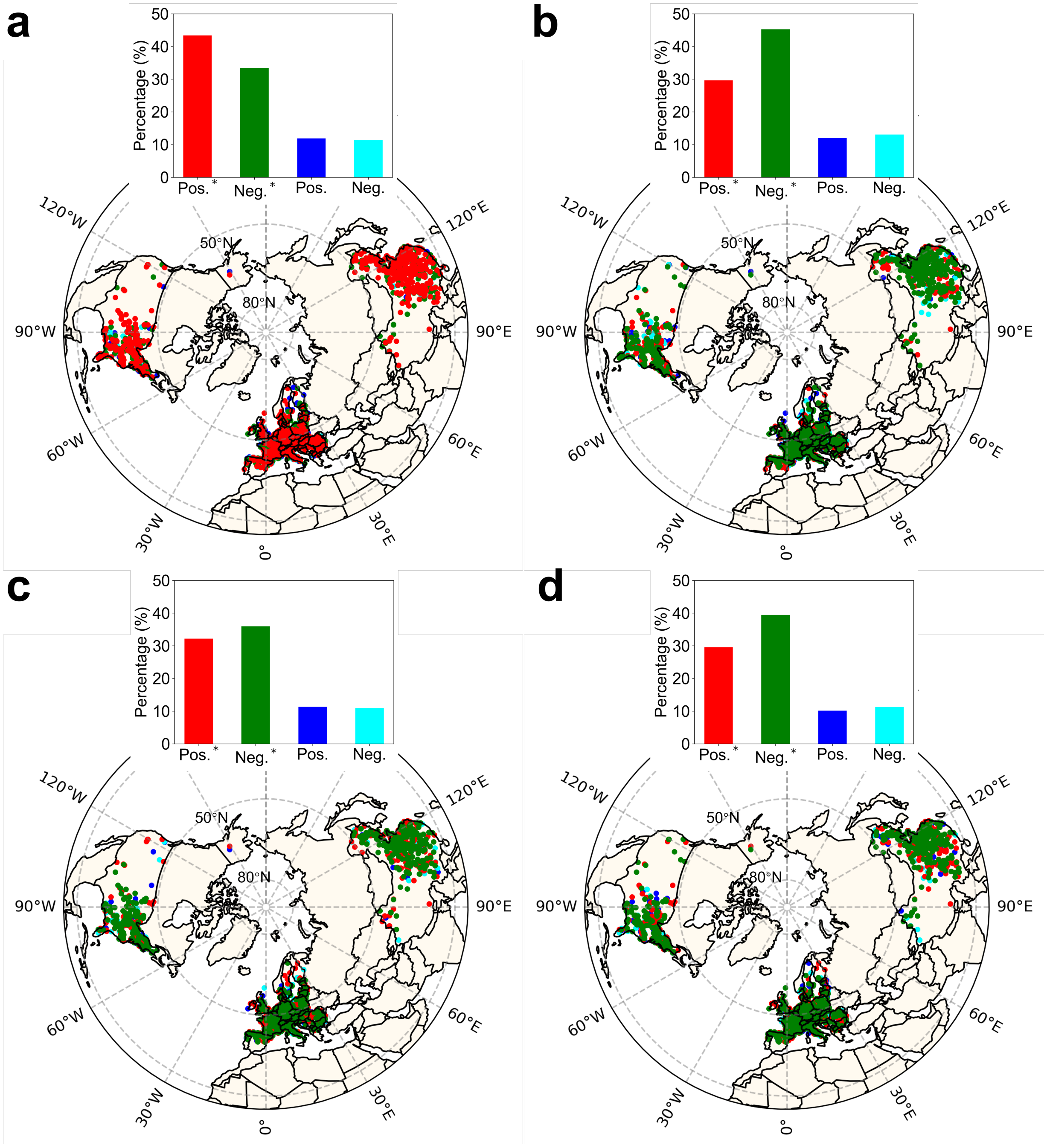

Figure S20. Spatial patterns of statistically significance at the 95% confidence level ($p < 0.05$) for partial correlation analysis between O_3 and SOS (**a**), EOS (**b**), EVI_{max} (**c**) and EVI_{area} (**d**). Colors represent the significant positive correlations (Pos.*), significant negative correlations (Neg.*), non-significant positive correlation (Pos.), and non-significant negative correlation (Neg.).

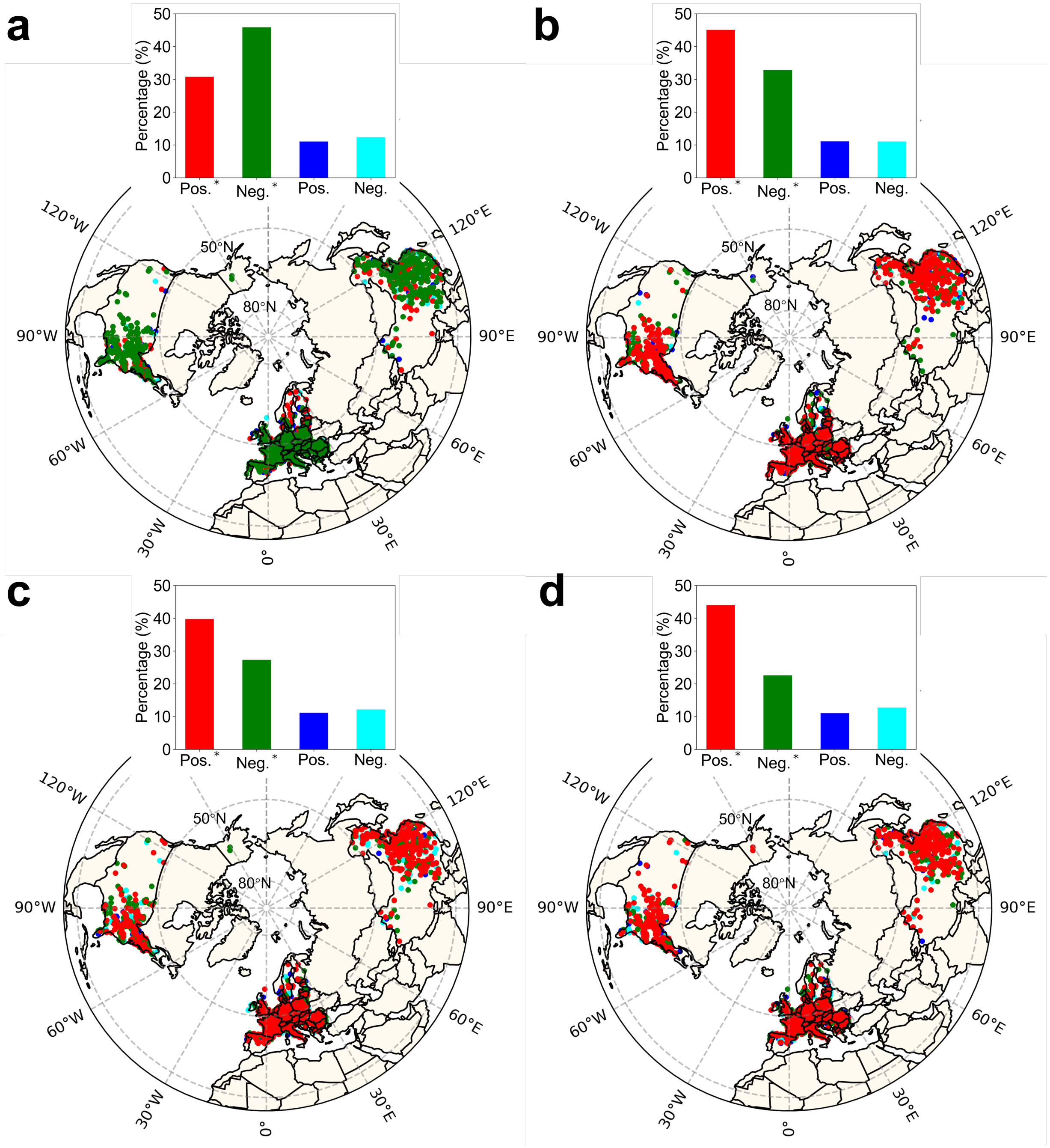

Figure S21. Spatial patterns of statistically significance at the 95% confidence level ($p < 0.05$) for partial correlation analysis between temperature and SOS (**a**), EOS (**b**), EVI_{max} (**c**) and EVI_{area} (**d**). Colors represent the significant positive correlations (Pos.*), significant negative correlations (Neg.*), non-significant positive correlation (Pos.), and non-significant negative correlation (Neg.).

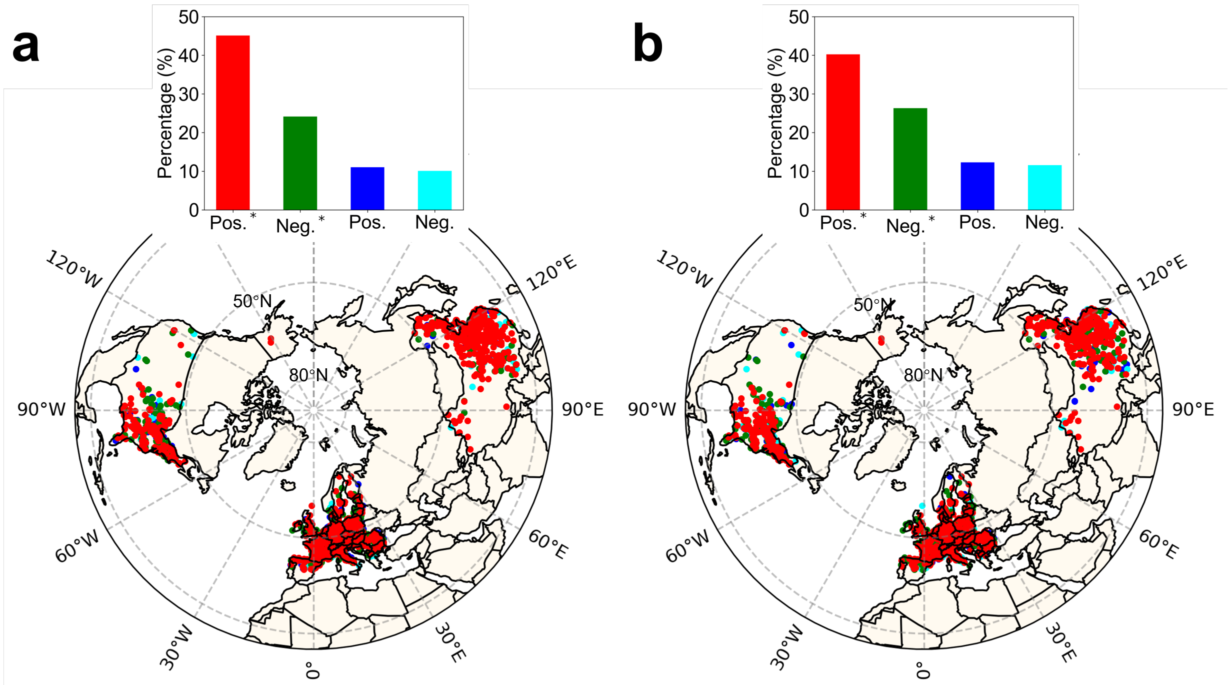

Figure S22. Spatial patterns of statistically significance at the 95% confidence level ($p < 0.05$) for partial correlation analysis between CO_2 and EVI_{max} (**a**) and EVI_{area} (**b**). Colors represent the significant positive correlations (Pos.*), significant negative correlations (Neg.*), non-significant positive correlation (Pos.), and non-significant negative correlation (Neg.).

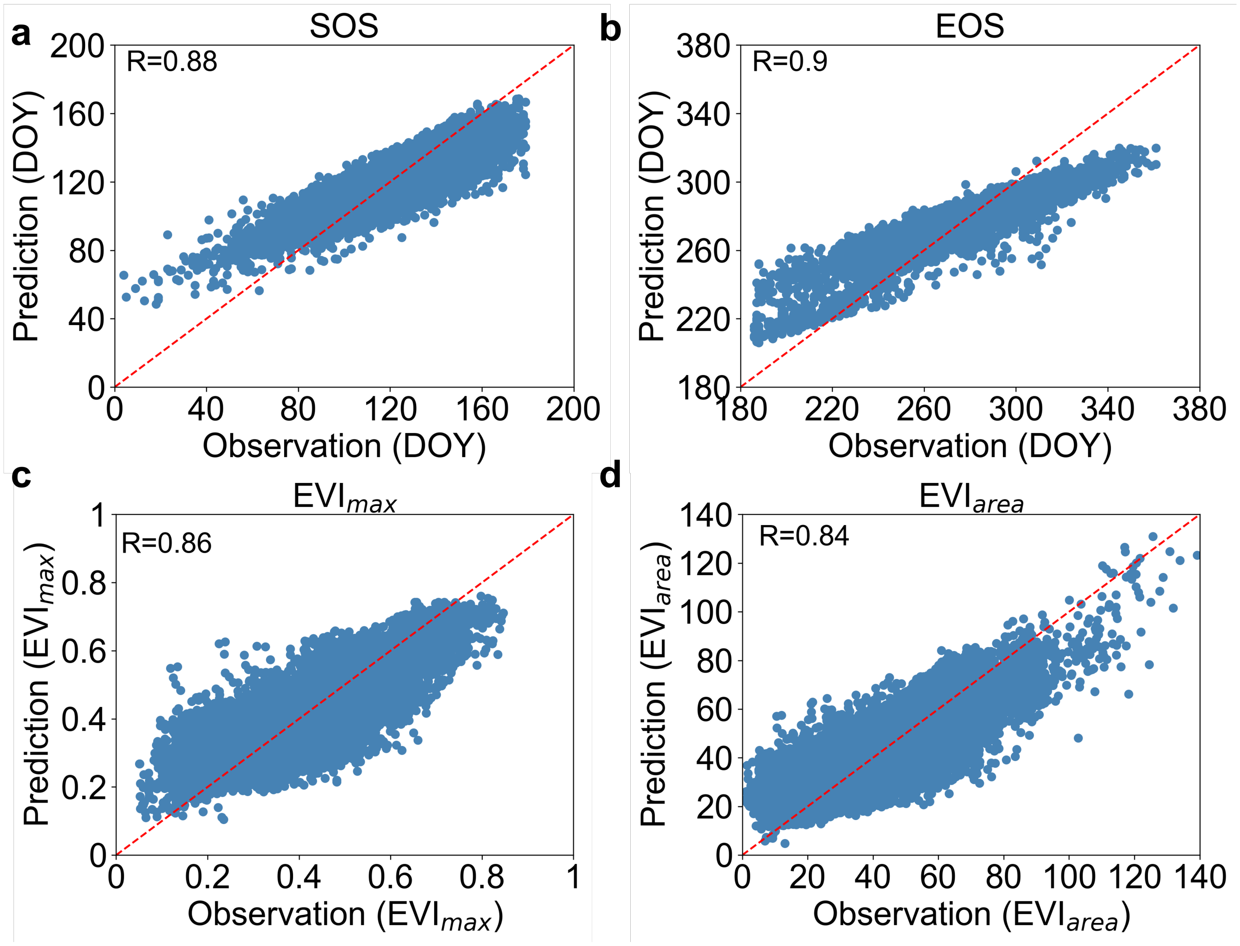

Figure S23. Model validation of LightGBM performance for SOS (**a**), EOS (**b**), EVI_{max} (**c**), and EVI_{area} (**d**) (n=24426, study period: US and EU: 2013-2022, CN: 2015-2022). R and dashed red 1:1 lines are shown in the figure.

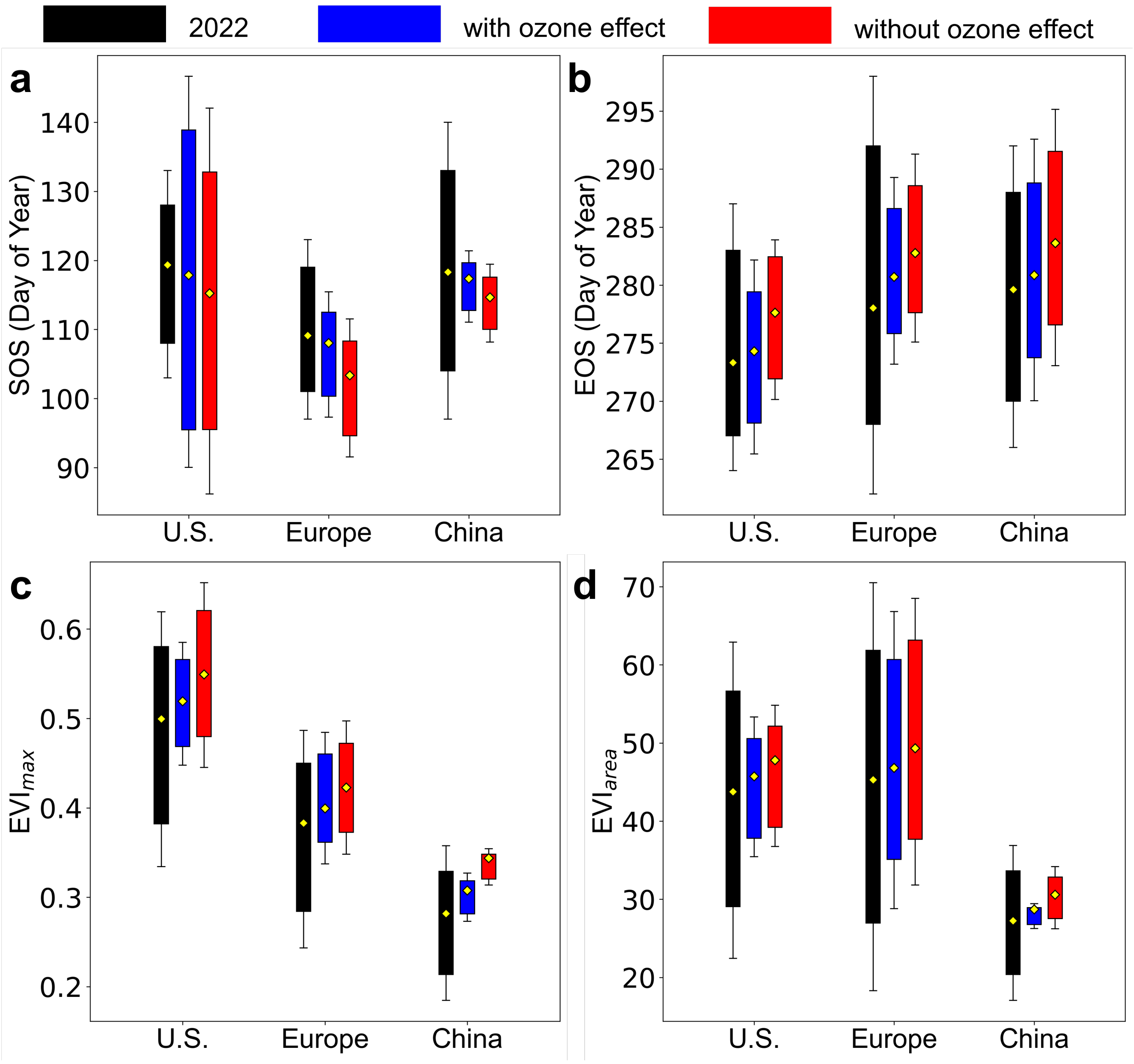

Figure S24. Prediction of SOS (**a**), EOS (**b**), EVI_{max} (**c**), EVI_{area} (**d**) for U.S., Europe, and China in 2050 under Shared Socioeconomic Pathway (SSP) 5-8.5. The black bar represents the observation in 2022. No-ozone effect (red bar) represents prediction with ozone concentration unchanged while ozone effect (blue bar) represents prediction using surface ozone data from CESM2.1.3 (see Methods). The diamonds indicate the average of partial correlations; box edges denote 25% and 75% quartiles. The whiskers represent 25% of the standard deviation.

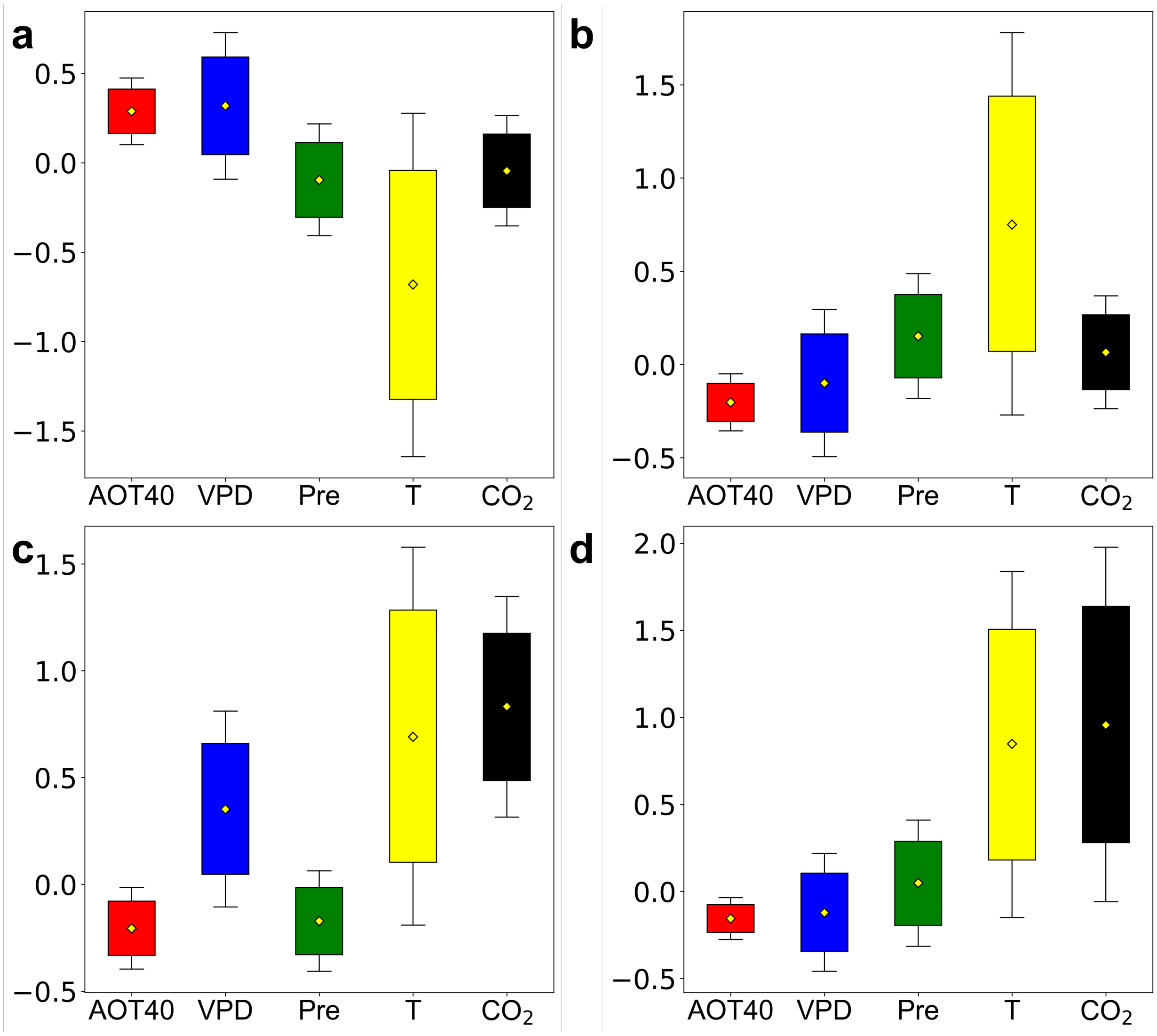

Figure S25. The average SHAP values for environmental variables for predicting SOS (**a**), EOS (**b**), and EVI_{max} (**c**), and EVI_{area} (**d**). Error bars represent the standard deviations of SHAP values across all sites. The diamonds indicate the average of partial correlations; box edges denote 25% and 75% quartiles; the whiskers represent 25% of the standard deviation.

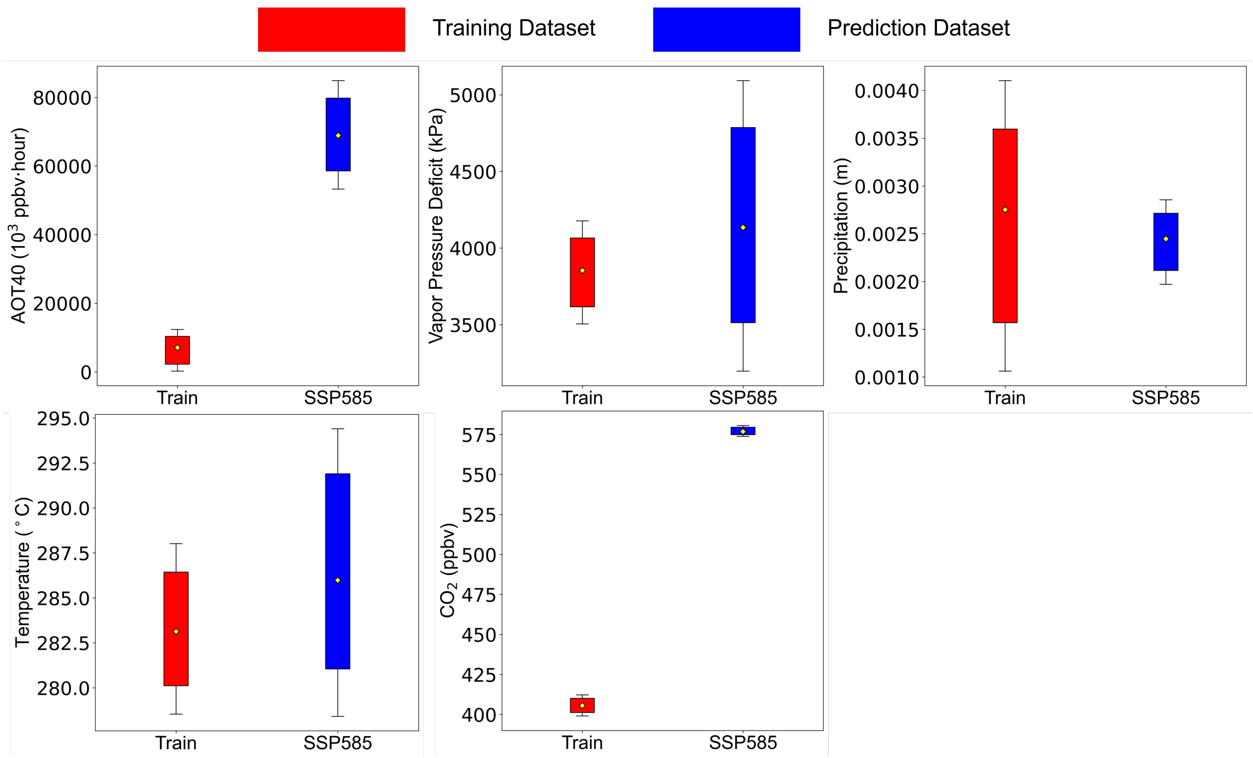

Figure S26. The distribution ranges of the variables of model input between the training data and prediction data in 2050 under Shared Socioeconomic Pathway (SSP) 5-8.5. The diamonds indicate the average of partial correlations; box edges denote 25% and 75% quartiles. The whiskers represent 25% of the standard deviation.
