# Supplementary material for: Ozone mitigates extended growing season and enhanced vegetation greenness driven by environmental change
Source: Nat Commun. 2026 Apr 20;17:5445. doi: 10.1038/s41467-026-71959-y (PMC13280477; doi:10.1038/s41467-026-71959-y)
Supplement: Supplementary file 3 — Reporting Summary [file 41467_2026_71959_MOESM3_ESM.pdf]

nature portfolio

Corresponding author(s): Lin Meng

Last updated by author(s): 3/5/2026

Reporting Summary

Nature Portfolio wishes to improve the reproducibility of the work that we publish. This form provides structure for consistency and transparency in reporting. For further information on Nature Portfolio policies, see our Editorial Policies and the Editorial Policy Checklist.

Statistics

For all statistical analyses, confirm that the following items are present in the figure legend, table legend, main text, or Methods section.

n/a	Confirmed	
[ ]	[x]	The exact sample size (*n*) for each experimental group/condition, given as a discrete number and unit of measurement
[x]	[ ]	A statement on whether measurements were taken from distinct samples or whether the same sample was measured repeatedly
[ ]	[x]	The statistical test(s) used AND whether they are one- or two-sided<br>*Only common tests should be described solely by name; describe more complex techniques in the Methods section.*
[ ]	[x]	A description of all covariates tested
[ ]	[x]	A description of any assumptions or corrections, such as tests of normality and adjustment for multiple comparisons
[ ]	[x]	A full description of the statistical parameters including central tendency (e.g. means) or other basic estimates (e.g. regression coefficient) AND variation (e.g. standard deviation) or associated estimates of uncertainty (e.g. confidence intervals)
[ ]	[x]	For null hypothesis testing, the test statistic (e.g. *F*, *t*, *r*) with confidence intervals, effect sizes, degrees of freedom and *P* value noted<br>*Give P values as exact values whenever suitable.*
[x]	[ ]	For Bayesian analysis, information on the choice of priors and Markov chain Monte Carlo settings
[x]	[ ]	For hierarchical and complex designs, identification of the appropriate level for tests and full reporting of outcomes
[ ]	[x]	Estimates of effect sizes (e.g. Cohen's *d*, Pearson's *r*), indicating how they were calculated

Our web collection on statistics for biologists contains articles on many of the points above.

Software and code

Policy information about availability of computer code

Data collection	No software/code is used in data collection.
Data analysis	Python v3.11 scripts are used in the data analysis described in the manuscript. All codes are available at https://doi.org/10.5281/zenodo.18826646

For manuscripts utilizing custom algorithms or software that are central to the research but not yet described in published literature, software must be made available to editors and reviewers. We strongly encourage code deposition in a community repository (e.g. GitHub). See the Nature Portfolio guidelines for submitting code & software for further information.

Data

Policy information about availability of data

All manuscripts must include a data availability statement. This statement should provide the following information, where applicable:

- Accession codes, unique identifiers, or web links for publicly available datasets
- A description of any restrictions on data availability
- For clinical datasets or third party data, please ensure that the statement adheres to our policy

All data used in this study are open-access data and can be directly downloaded through the websites described in the method section of the paper.

Research involving human participants, their data, or biological material

Policy information about studies with human participants or human data. See also policy information about sex, gender (identity/presentation), and sexual orientation and race, ethnicity and racism.

Reporting on sex and gender	N/A
Reporting on race, ethnicity, or other socially relevant groupings	N/A
Population characteristics	N/A
Recruitment	N/A
Ethics oversight	N/A

Note that full information on the approval of the study protocol must also be provided in the manuscript.

Field-specific reporting

Please select the one below that is the best fit for your research. If you are not sure, read the appropriate sections before making your selection.

☐ Life sciences ☐ Behavioural & social sciences ☒ Ecological, evolutionary & environmental sciences

For a reference copy of the document with all sections, see nature.com/documents/nr-reporting-summary-flat.pdf

Ecological, evolutionary & environmental sciences study design

All studies must disclose on these points even when the disclosure is negative.

Study description	We used multiple ground and satellite observations to investigate the effects of ozone on vegetation phenology and greenness in spatial and temporal scales over United States, Europe, and China for the past decade. We further predicted the future effects of ozone on vegetation phenology and greenness under future change scenarios.
Research sample	We used ground based ozone observations data from the United States Environmental Protection Agency, the European Environment Agency and the China National Environmental Monitoring Centre network. We extracted vegetation data and climate data for these ozone sites and conducted analysis for these sites.
Sampling strategy	No statistical methods were used to predetermine sample size. We used as much data as possible after filtering the data according to our criteria.
Data collection	All data used in this study are open-access data and included in the paper and/or the Supplementary Material. The specific links for datasets used in this study can be found in Material and Methods.
Timing and spatial scale	The ground-based ozone data used in this analysis was from 2013 to 2022 in Europe, and U.S, and from 2015 to 2022 in China. The vegetation phenology data, ERA5 data, and SNDRAQIL3CMCCP XCO2 datasets were from 2013 to 2022. The Global land 1° Mapping-XCO2 dataset was provided from 2013 to 2020. The CMIP6 model simulations data is provided from 2023 to 2050. Spatial scale is global scale including U.S., Europe, and China.
Data exclusions	We selected ozone sites in the following land cover types: evergreen needleleaf forests, evergreen broadleaf forests, deciduous needleleaf forests, deciduous broadleaf forests, mixed forests, shrublands, savannas, and urban lands. To ensure the data quality, we remove outlier data in the following steps: (a) convert all hourly measurement data into Z scores, (b) remove the data if (1) Zi is larger or smaller than the previous value Zi-1 by 9 (|Zi-Zi-1|>9); or (2) the absolute value of Zi is greater than 4 (|Zi|>4); or (3) the ratio of Zi to the third-order center moving average is greater than 2 ((3Z_i)/(Z_(i-1)+Z_i+Z_(i+1))>2), where i represents the i-th hourly measurement data.
Reproducibility	The results are fully reproducible by using the data and codes provided and by following the description in the Methods section.
Randomization	Randomization is not applicable in this study.
Blinding	Blinding was not relevant to our study.

Did the study involve field work? ☐ Yes ☒ No

Reporting for specific materials, systems and methods

We require information from authors about some types of materials, experimental systems and methods used in many studies. Here, indicate whether each material, system or method listed is relevant to your study. If you are not sure if a list item applies to your research, read the appropriate section before selecting a response.

Materials & experimental systems

n/a	Involved in the study
☒	☐ Antibodies
☒	☐ Eukaryotic cell lines
☒	☐ Palaeontology and archaeology
☒	☐ Animals and other organisms
☒	☐ Clinical data
☒	☐ Dual use research of concern
☒	☐ Plants

Methods

n/a	Involved in the study
☒	☐ ChIP-seq
☒	☐ Flow cytometry
☒	☐ MRI-based neuroimaging

Plants

Seed stocks

Report on the source of all seed stocks or other plant material used. If applicable, state the seed stock centre and catalogue number. If plant specimens were collected from the field, describe the collection location, date and sampling procedures.

Novel plant genotypes

Describe the methods by which all novel plant genotypes were produced. This includes those generated by transgenic approaches, gene editing, chemical/radiation-based mutagenesis and hybridization. For transgenic lines, describe the transformation method, the number of independent lines analyzed and the generation upon which experiments were performed. For gene-edited lines, describe the editor used, the endogenous sequence targeted for editing, the targeting guide RNA sequence (if applicable) and how the editor was applied.

Authentication

Describe any authentication procedures for each seed stock used or novel genotype generated. Describe any experiments used to assess the effect of a mutation and, where applicable, how potential secondary effects (e.g. second site T-DNA insertions, mosiacism, off-target gene editing) were examined.
