# Supplementary material for: Ozone mitigates extended growing season and enhanced vegetation greenness driven by environmental change
Source: Nat Commun. 2026 Apr 20;17:5445. doi: 10.1038/s41467-026-71959-y (PMC13280477; doi:10.1038/s41467-026-71959-y)
Supplement: Supplementary file 2 — Peer Review file [file 41467_2026_71959_MOESM2_ESM.pdf]

nature portfolio

Peer Review File

Ozone mitigates extended growing season and enhanced vegetation greenness driven by environmental change

Corresponding Author: Professor Lin Meng

This file contains all reviewer reports in order by version, followed by all author rebuttals in order by version.

Version 0:

Reviewer comments:

Reviewer #1

(Remarks to the Author)
Yin et al. “Ozone mitigates extended growing season and enhanced vegetation greenness driven by climate change”

This study assesses the impact of surface ozone on global vegetation phenology. The findings indicate that ozone delays the start of season (SOS) and advances the end of season (EOS) in highly polluted regions such as the United States, Europe, and China. To some extent, this effect offsets the extension of the growing season driven by global warming. While the study presents novel insights, it has substantial limitations in terms of data, methodology, and mechanistic explanations. Therefore, I do not recommend that this article be accepted. My specific comments are as follows:

First, the data used in analyses are not complete. The study relies on only one set of satellite-derived data. However, previous research has shown that different remote sensing products yield substantial uncertainties in different phenological data (https://essd.copernicus.org/preprints/essd-2024-225/). Multiple satellite datasets should be used to verify the robustness of the findings. Regarding data coverage, the study includes only seven years of data for China and just ten years for North America and Europe. The short length of these datasets raises concerns about statistical significance due to the limited sample size.

Second, the methods applied are not reasonable. The study primarily employs linear correlations, which are inadequate for capturing nonlinear relationships between variables and fail to account for collinearity among factors. Although partial correlation analysis was used, collinearity issues cannot be entirely eliminated. Additionally, in future projections, the study assumes five future scenarios with fixed ozone concentration change rates. This assumption is problematic because ozone concentration changes vary across regions under different scenarios. More importantly, ozone concentration may exhibit a nonlinear response to the same level of anthropogenic emission reductions under the different chemical background.

Third, the conclusions are not reliable. The study relies on correlation analysis, yet the correlation coefficients between phenological parameters and both temperature and ozone are very low. For example, correlation coefficients between AOT40 and SOS were only 0.15 ± 0.04 in the U.S., 0.06 ± 0.02 in Europe, and 0.10 ± 0.03 in China (Lines 149-150). Spatial distribution maps of Fig. 3a reveal a highly scattered response in China, lacking a clear and significant pattern. Moreover, the study claims that ozone leads to a decline in EVImax (Fig. 3e). However, in recent years, ozone concentration in China has been increasing (Li et al., 2020), while EVImax and vegetation greenness have also shown a significant upward trend (Chen et al., 2019). If a simple correlation analysis were applied, these two variables would be expected to show a positive correlation, contradicting the study’s conclusions.

Finally, mechanistic explanations are inadequate. The study’s key finding is that ozone shortens the growing season, but it does not provide direct evidence to support this conclusion. Instead, it mainly cites previous studies. The discussion section is largely devoted to explaining ozone’s nonlinear responses while neglecting the nonlinear interactions between ozone and vegetation. Furthermore, the study fails to consider differences in responses to ozone among vegetation types, as well as regional variations in these responses and their underlying mechanisms.

Reference
Chen C, et al. China and India lead in greening of the world through land-use management. Nat Sustain 2, 122-129 (2019).
Li K, Jacob DJ, Shen L, Lu X, De Smedt I, Liao H. Increases in surface ozone pollution in China from 2013 to 2019:

anthropogenic and meteorological influences. Atmos Chem Phys 20, 11423-11433 (2020).

(Remarks on code availability)

Reviewer #2

(Remarks to the Author)
This paper has the noteworthy results of showing, through a partial correlation analyses, the influence of tropospheric ozone levels on continental scale trends in vegetation growing season parameters as well as integrated growing season and peak vegetation productivity. The hypothesis posed is that ozone levels will delay start of season, while advancing senescence or end of growing season, yielding an overall shortening of the growing season and decline in vegetation productivity. Many local scale studies have also shown similar negative impacts due to surface ozone over specific areas, but this is a first study to look at this across 3 continents and the authors use of partial correlation analyses seeks to separate the ozone influence from that of CO_2 concentrations and warming temperatures.

There are some apparent weaknesses which may require further analysis and evidence confirm and better understand the results - in order for the study to be convincing.

1. Only three factors (temperature, CO_2, ozone) on phenology and productivity are analysed. These are the 2 dominant climate change drivers of temperature increases and CO_2 increases, with ozone added as the third driver of interest. However, there are other climatic drivers known to affect phenology, in particular precipitation/ water availability and VPD which are ignored throughout the study. Water availability, in particular, is likely the dominant driver of phenology in some regions (e.g. arid US, Mediterranean Europe). Other non-climatic factors such as nitrogen deposition, PM2.5 and pollutants (in China) are also not taken into account. By ignoring these factors, there is a risk that changes in phenology are mis-attributed to ozone effects.

2. Although the US and Europe had 3-fold lower ozone levels than China, ozone in US/ EU appeared to result in stronger effects on vegetation than China. At least this is based on the strengths of partial correlation analyses, where we see stronger delays in SOS and advances in EOS; as well stronger negative correlations with peak and integrated satellite EVI at the USA/ EU sites relative to China. Maybe a fixed set of latitudes are needed?

3. In addition to the partial correlation analyses of trends, the authors could have provided indications of the slope of the trends, to show, for example, the delay in SOS days/ decade attributed to ozone and quantify the offset to phenology attributed to temperature in each continent. It is possible the time series analyzed, 8-10 years, is insufficient to draw these conclusions. However, the authors could have used the equivalent dataset and products derived from MODIS satellite sensor and have a 24+ years time series results of changing phenologies. It's also curious why the current VIIRS satellite analyses uses different years for China (2015-22) than the other continents (2013-2022).

4. Ozone patterns vary highly across latitudes (showing mid-latitude peaks), yet in all figures and discussion, latitude information is missing. In particular Fig. 2 lacks both scale and locational information, and the 3 continents are displayed side by side, as if they are latitudinally aligned, which they are not. It would be very informative to additionally compare results within aligned latitudinal bands.

5. Aside, from latitude, ozone effects are highly variable across sites (Figure 3). The existing background variability would need to be explored in more detail. For example. what factors cause such a spatially variable response to ozone as shown in Figure 3? How are these effects and their uncertainties handled from a statistical point of view. For example, in line 168 the ozone effects on SOS are given as 0.09 +/- 0.02, which implies a high certainty of the effect being positive, which seems to contradict the highly variable effects shown in Figure 3a. The uncertainty metric is given as S.D. in line 150, yet it is clear that S.D. is higher than 0.02 in Figure 3a. As such, the confidence in the results seem to be overstated.

6. It would add much. value to this study to further select targeted areas that show more explicitly phenology change patterns (as in Figure 1) across the sources of variability, including vegetation type, latitude, urban/ rural classes, etc. Its' quite difficult to follow the authors observed interpretation from very small global maps.

7. The future projections are of purely statistical nature and assume that temperature and CO_2 effects in the future are the same as they are in the past. This is a strong assumption as effects are non-linear (e.g. biological temperature responses show an optimum, CO_2 effects saturate). In addition, other climate change factors, in particular water and nutrient dynamics, are ignored. I suggest running a process-based model such as CLM into the future. This would allow to a) isolate ozone effects and b) take all major climate change factors and their non-linearities into account.

Detailed comments:
Abstract:
- The statement in Abstract "Using multiple ground and satellite observations", could be misinterpreted as several ground and satellite datasets. More accurately, it would be clearer to write "Using ground observations of ozone and satellite observations".
- The motivation for this study needs to be clearer (Lines 23-24). Is the gap in knowledge that ozone effects on vegetation

phenology are unknown or is it that large scale ozone effects are unknown?

Results:
Figure 2a-c: time period of observations should be given in Figure caption.
Figure 2d: maps are very small and difficult to read
Most Figures- missing scale and latitude/long information.
Figure 2d results (line 152). Authors write "This indicates elevated ozone is associated with the lengthening of growing season by shifting both spring and autumn phenology." This runs counter to the rest of the study.

(Remarks on code availability)

Reviewer #3

(Remarks to the Author)
I co-reviewed this manuscript with one of the reviewers who provided the listed reports. This is part of the Nature Communications initiative to facilitate training in peer review and to provide appropriate recognition for Early Career Researchers who co-review manuscripts.

(Remarks on code availability)

Reviewer #4

(Remarks to the Author)
The paper “Ozone mitigates extended growing season and enhanced vegetation greenness driven by climate change” by Yin et al submitted to Nature Communications investigates the influence of increasing surface ozone concentration on vegetation phenology and greenness. Main results reported indicate that higher ozone concentration will mitigate climate change impacts expected to lengthen the growing season by decreasing the season up to 10 days and also reducing the accumulated enhanced vegetation index. The paper reads well and has a clear message, that brings the management of surface ozone concentration to an urgent level.

My review focused on the methods used for the analyses. The authors indicate they have done a “partial correlation analyses”, which is an interesting method to use in this case aiming to tease out the effect of the variables of interest (e.g., ozone) by controlling the effect of other variables (e.g., temperature, in this case).
There are a few points around the methods and analyses used that would be good to have clarified in the manuscript:

1) Could the authors confirm if equation 2 is correctly written? Shouldn’t the second part of the numerator be “- R(X,Z) R(Y,Z)” instead of “- R(X,Y) R(Y,Z)”?

2) More detail on the difference of the models for the spatial and temporal components would be useful, for example, how did the authors deal with correlation between the variables that were not being controlled for in each of these analyses?

3) The phenological prediction was based on MLR, which includes the same assumptions as simple linear models, however none of the checks on these assumptions was reported in the manuscript. Adding a summary of these checks would be beneficial to aid interpretation of the model results.

4) The authors present equation 3 for the MLR stating it represents a linear combination of multiple independent variables, however they never confirm independence of the variables included as predictors in this model. For example, are temperatures for SOS and EOV correlated? OR the EVImax with EVIarea? A statement about the correlations (or a table with the results in SI) could be beneficial

5) The prediction into the future is likely to be the result of extrapolation but no information is provided in the Methods. Although it is clearly a problem for the future scenarios and also the reason why multiple scenarios are tested, it would still be good have an idea of how far the variables used as predictors are from the ranges of values used in the training models.

6) A statement clearly discussing the results for the predictors across scenarios should clearly explain the results in different directions and in which cases they happen (e.g., many of the figures in SI show increasing and decreasing trends, however the manuscript focus on a single trend: ozone concentrations will reduce the expected effects of climate change.

(Remarks on code availability)

Version 1:

Reviewer comments:

Reviewer #1

(Remarks to the Author)
The paper has been revised in accordance with the referees' comments, incorporating additional satellite data and statistical methods (e.g., LightGBM). I have no further comments and agree with the acceptance of the study.

(Remarks on code availability)

Reviewer #2

(Remarks to the Author)
My ECR co-reviewer and myself wish to acknowledge the significant revisions and new analyses conducted by the authors which have resulted in a much improved manuscript with results that are very convincing. The authors have now included significant co-variates into their partial correlation analyses, which have maintained the initial statistically relevant ozone impacts in both the spatial and temporal domains. The few variables that could not be integrated into the analyses, such N-deposition, were properly acknowledged as limitations in the Discussion section, making this section more interesting for future studies/ thoughts.
We are also now satisfied with the uncertainty analyses and other responses to our comments.

In fact, the only remaining concern is around the response to our comment #6 that focuses on more detailed targeted areas to confirm findings. The paper is overall written in the style of a global analyses on vegetation phenology, however, we feel this may be quite misleading and lead to misinterpretations by many, since as we know most ozone networks and sparsely located and prioritize urban and peri-urban locations only because population exposure is highest there and thus most relevant for health risk assessments.
The authors mention that more ground ozone measures are needed in rural areas, however, in the authors response to our comment 6, it was pointed out that 77% of the sites used in this paper were urban sites. Of the remaining 23%, the majority of these are most likely very close to urban centers? The spatial cluster maps give the impression and exaggerated wider coverage. The authors do mention that more sites outside urban areas are needed, but only at the end of the Discussion. We feel this is needs to be stated upfront and probably in the abstract. It does not deter from the significant findings of this study - it merely constrains the results to where they belong - urban vegetation and their adjacent ecosystem surroundings. The authors could have selected the 'most' remote ground ozone sites to compare against the urban sites, if they really wanted to claim a global analyses - or used satellites.
We think urban vegetation also has different temperature and CO2 regimes, in contrast to rural and remote areas.

The authors did mention satellite-based ozone products in Discussion and noted their limitations. However, it could make better discussion to say ground-calibrated ozone fused with satellite ozone can enable spatial extrapolations and provide the means to assess phenology- ozone relationships distant from urban areas (this is done with EC flux towers and satellites). Many studies are being published with ozone and smoke from remote fires. Current satellites, as TROPOMI, provide global, repeated coverage, making it possible to track broad changes in tropospheric ozone that are not feasible with sparse ground-based networks alone. This makes for more interesting,, positive discussions (we're not asking for more analyses).

Minor comments: the authors should provide actual url links to the ozone sites/ data, rather than links to the agencies. I click on the US- EPA ground ozone link and it took be to the homepage of EPA. I searched for ozone monitoring and it gave 500,000 sites?

(Remarks on code availability)

Reviewer #3

(Remarks to the Author)
I co-reviewed this manuscript with one of the reviewers who provided the listed reports. This is part of the Nature Communications initiative to facilitate training in peer review and to provide appropriate recognition for Early Career Researchers who co-review manuscripts.

(Remarks on code availability)

Reviewer #4

(Remarks to the Author)
The authors have done a huge amount of work in the revision of this manuscript with many additions alleviating most of the concerns in the comments I provided. I particularly liked the addition of LightGBM and SHAP.
My comments were therefore mostly addressed.

The only point missing is a quick visual representation of the differences in the data used for model development and for prediction into the future (e.g., boxplots showing how much the future data differs from the data used to train the model). Together with a brief comment on the results, this representation would assist assessing the level of extrapolation included in the results.

(Remarks on code availability)

Open Access This Peer Review File is licensed under a Creative Commons Attribution 4.0 International License, which permits use, sharing, adaptation, distribution and reproduction in any medium or format, as long as you give appropriate credit to the original author(s) and the source, provide a link to the Creative Commons license, and indicate if changes were made.
In cases where reviewers are anonymous, credit should be given to 'Anonymous Referee' and the source.
The images or other third party material in this Peer Review File are included in the article's Creative Commons license, unless indicated otherwise in a credit line to the material. If material is not included in the article's Creative Commons license and your intended use is not permitted by statutory regulation or exceeds the permitted use, you will need to obtain permission directly from the copyright holder.
To view a copy of this license, visit https://creativecommons.org/licenses/by/4.0/

REVIEWER COMMENTS

Reviewer #1 (Remarks to the Author):

Yin et al. "Ozone mitigates extended growing season and enhanced vegetation greenness driven by climate change"

This study assesses the impact of surface ozone on global vegetation phenology. The findings indicate that ozone delays the start of season (SOS) and advances the end of season (EOS) in highly polluted regions such as the United States, Europe, and China. To some extent, this effect offsets the extension of the growing season driven by global warming. While the study presents novel insights, it has substantial limitations in terms of data, methodology, and mechanistic explanations. Therefore, I do not recommend that this article be accepted. My specific comments are as follows:

Response: Thanks for your thorough and constructive comments. We appreciate your evaluation of our study and your recognition of its novel insights. Below, we provide point-by-point responses to address your concerns. We have revised the manuscript to clarify and strengthen data selection, methodological decisions, and mechanistic explanations.

1. First, the data used in analyses are not complete. The study relies on only one set of satellite-derived data. However, previous research has shown that different remote sensing products yield substantial uncertainties in different phenological data (https://essd.copernicus.org/preprints/essd-2024-225/). Multiple satellite datasets should be used to verify the robustness of the findings. Regarding data coverage, the study includes only seven years of data for China and just ten years for North America and Europe. The short length of these datasets raises concerns about statistical significance due to the limited sample size.

Response: Thanks for your comments. Our initial analysis is based on VIIRS Global Land Surface Phenology dataset, which provides spatially consistent, well-calibrated, and up-to-date estimates of phenological transitions across global vegetated regions. This dataset was selected because of its strong biophysical linkage to land surface reflectance and vegetation activity, and its increasing adoption in phenological studies focused on ecosystem responses to environmental change (Dutra et al., 2025; Zhang et al., 2017, 2018). In line with your suggestion, we have now incorporated the MODIS phenology product and reproduced our main analyses, obtaining consistent results across both datasets (Fig. S8). Also, we already used two CO_2 datasets (Fig. S7) and two ozone metrics (AOT40 and average ozone concentrations, Fig. S6), which all shows consistent patterns (see Lines 211-236). These cross-validations strengthen confidence in the robustness of our findings.

Regarding the temporal coverage, the study period was constrained primarily by the availability of reliable, spatially extensive ground-based surface ozone measurements. In China, nationally gridded surface ozone data based on in situ observations are only available from 2015 onwards. Earlier ozone records, where available, are sparse, inconsistent, or localized, making it infeasible to construct a robust spatiotemporal dataset for national-scale phenology-ozone analyses. The comparability of the data is affected by methodological inconsistencies in collection and processing prior to 2013 in Europe and U.S.A. Subsequent data adhere to a more harmonized and rigorous standard, significantly improving their robustness for cross-national analysis. We conducted an extensive review of alternative ozone products, including reanalysis and model-based estimates (e.g., Liu et al., 2022), but found they either lacked observational grounding or did not provide adequate spatial and temporal resolution for our purpose of study.

To mitigate the limitations of relatively short time series and address other comments, we revised our approach to leverage additional machine learning techniques (see details in the response #2 below), emphasizing spatiotemporal comparison of regional differences, which reduce the impact of sample size constraints on the results. We also cross-checked results across different satellite products and individual vegetation type. The consistency of the ozone-phenology signal supports the robustness of the observed patterns. We have now revised the Method section in the manuscript to clarify the rationale for selecting phenology products, and update model description (see lines 617-626, lines 793-833).

Liu, X., Zhu, Y., Xue, L., Desai, A. R., and Wang, H.: Cluster-Enhanced Ensemble Learning for Mapping Global Monthly Surface Ozone From 2003 to 2019, *Geophysical Research Letters,* 49, e2022GL097947, https://doi.org/10.1029/2022GL097947, 2022.

2. Second, the methods applied are not reasonable. The study primarily employs linear correlations, which are inadequate for capturing nonlinear relationships between variables and fail to account for collinearity among factors. Although partial correlation analysis was used, collinearity issues cannot be entirely eliminated. Additionally, in future projections, the study assumes five future scenarios with fixed ozone concentration change rates. This assumption is problematic because ozone concentration changes vary across regions under different scenarios. More importantly, ozone concentration may exhibit a nonlinear response to the same level of anthropogenic emission reductions under the different chemical background.

Response: Thanks for your comments, which has led us to significantly revise and strengthen our analytical approach in the revised manuscript. To address the limitation of linear correlation and collinearity, the revised manuscript now adds LightGBM (Light Gradient Boosting Machine), an advanced machine learning algorithm, and SHAP (SHapley Additive exPlanations) that interpret the predictions of the LightGBM model on changes in vegetation phenology and greenness under changing ozone scenarios. We also incorporated precipitation and VPD as additional covariates in the model, as suggested by reviewer in other comments. The tree-like architecture of LightGBM inherently handles collinearity by selecting the most informative features during node splitting, thereby eliminating the bias caused by correlation between variables. Unlike linear models, it automatically captures complex nonlinear interactions without requiring predefined functional forms, making it ideally suited for modeling ozone's multifaceted relationships with environmental drivers. To ensure model robustness and reproducibility, we implemented rigorous 10-fold cross-validation, hyperparameter sensitivity tests, and bootstrap-based uncertainty quantification. The results show that LightGBM achieves high performance and exhibits strong resistance to overfitting (Fig. S23).

In terms of future ozone scenario assumptions, we have replaced the earlier simplified fixed-rate scenarios with spatially explicit, scenario-dependent ozone projections simulated using the Community Earth System Model version 2 (CESM2) (Bhattarai et al. 2024). Anthropogenic and biomass burning emissions followed the CMIP6 inventory, while future scenarios of land use, greenhouse gases, and short-lived species were prescribed from Shared Socioeconomic Pathways (SSPs). These data incorporate region-specific ozone responses under different SSP scenarios while accounting for nonlinear chemical behavior of ozone and its sensitivity to emissions under different

baseline atmospheric conditions. This advancement ensures our projections reflect spatial heterogeneities and scenario-dependent chemical regimes.

All methodological revisions have been thoroughly documented in the updated Methods section (lines 708-735, lines 793-833), with corresponding updates to results presented in the subsection ("Vegetation changes under future ozone scenarios", lines 306-339). We believe these revisions address the methodological concerns raised and significantly improve the rigor of the study's analytical framework.

Bhattarai, H., Tai, A. P. K., Val Martin, M., and Yung, D. H. Y.: Impacts of changes in climate, land use, and emissions on global ozone air quality by mid-21st century following selected Shared Socioeconomic Pathways, *Science of The Total Environment*, 906, 167759, https://doi.org/10.1016/j.scitotenv.2023.167759, 2024.

3. Third, the conclusions are not reliable. The study relies on correlation analysis, yet the correlation coefficients between phenological parameters and both temperature and ozone are very low. For example, correlation coefficients between AOT40 and SOS were only 0.15 ± 0.04 in the U.S., 0.06 ± 0.02 in Europe, and 0.10 ± 0.03 in China (Lines 149-150). Spatial distribution maps of Fig. 3a reveal a highly scattered response in China, lacking a clear and significant pattern. Moreover, the study claims that ozone leads to a decline in EVImax (Fig. 3e). However, in recent years, ozone concentration in China has been increasing (Li et al., 2020), while EVImax and vegetation greenness have also shown a significant upward trend (Chen et al., 2019). If a simple correlation analysis were applied, these two variables would be expected to show a positive correlation, contradicting the study's conclusions.

Reference

Chen C, et al. China and India lead in greening of the world through land-use management. Nat Sustain 2, 122-129 (2019).
Li K, Jacob DJ, Shen L, Lu X, De Smedt I, Liao H. Increases in surface ozone pollution in China from 2013 to 2019: anthropogenic and meteorological influences. Atmos Chem Phys 20, 11423-11433 (2020).

Response: Thanks for raising these important points. We have addressed these points through additional analyses and clarifications in the revised manuscript:

(1) On the strength of ozone–phenology relationships:
We acknowledge that the observed correlation coefficients between ozone exposure (AOT40) and phenological parameters are relatively modest in magnitude. However, statistical significance was consistently observed across regions ($p < 0.05$), as shown in the updated partial correlation results (Fig. 2). Importantly, the modest effect sizes reflect the reality that ozone is not the dominant driver of phenological change, rather, ozone operates as a secondary but detectable stressor, consistent with previous studies showing that temperature and photoperiod remain the primary phenological controls at regional scales. As the field progresses toward understanding multi-factorial controls on phenology, identifying and quantifying the contributions of secondary stressors like ozone becomes

increasingly important. Our conclusions do not overstate ozone's role; rather, they emphasize its statistically robust yet relatively small effect size, particularly when evaluated alongside other environmental drivers such as temperature, precipitation, and VPD. We clarified this in the manuscript (lines 250-254, lines 381-384).

(2) On spatial heterogeneity in China:
We do not interpret the spatial heterogeneity in China as an absence of meaningful signal. Instead, it reflects the spatial complexity of ozone–phenology interactions in China, which vary according to local vegetation type, urbanization level, meteorology, and background pollution levels. In the revised manuscript, we now explicitly discuss these regional complexities (lines 450-461), and we have retained statistically significant ozone-phenology relationships for China at the national level.

(3) On the relationship between ozone and vegetation greenness:
The reviewer correctly notes that both ozone concentrations and vegetation greenness have increased in China in recent years. However, our study does not rely on simple bivariate trends. Instead, we use partial correlation to isolate the role of ozone while controlling for confounding influences such as temperature, CO_2, VPD, and precipitation. The revised Figure 2 shows ozone has a small but significant negative relationship with EVI_{max} (partial correlations: $R_{AOT40\text{-}EVImax}$: -0.11 ± 0.02 (U.S.), $p < 0.05$, -0.03 ± 0.01 (Europe), $p < 0.05$, and -0.10 ± 0.02 (China), $p < 0.05$) when other factors are held constant. However, this negative ozone effect is substantially outweighed by the strong positive effects of rising CO_2 and temperature shown in our analysis (Figs. 2, 3, S6, S11, and S25). Thus, while ozone exerts a modest suppressive influence, the net trend in EVImax remains positive due to dominant climate and CO_2 drivers. This is consistent with Chen et al. (2019) and Li et al. (2020). In other words, while ozone imposes a stress, it does not negate the broader trend of greening; rather, it may partially offset it. We have expanded the Discussion section (lines 450-461) to clearly explain this point and emphasize that our findings do not contradict recent observations of greening in China. Instead, they highlight that ozone is a growing constraint on vegetation productivity, especially in urban or industrial regions, and should be considered alongside other environmental drivers.

4. Finally, mechanistic explanations are inadequate. The study's key finding is that ozone shortens the growing season, but it does not provide direct evidence to support this conclusion. Instead, it mainly cites previous studies. The discussion section is largely devoted to explaining ozone's nonlinear responses while neglecting the nonlinear interactions between ozone and vegetation. Furthermore, the study fails to consider differences in responses to ozone among vegetation types, as well as regional variations in these responses and their underlying mechanisms.
Response: Thanks for your thoughtful comments, which has helped us clarify and strengthen the mechanistic foundation of our study. First, the goal of this study is not to provide new mechanistic experiments, but rather to extend the existing body of plant physiological research by testing whether the ozone effect is detectable and significant at large spatial scales. We provide robust empirical, region-specific evidence of ozone's vegetation impacts (Figs. 2-3), and we consider these analyses to provide direct empirical support for the conclusion that ozone exposure shortens the growing season. We then place these new findings in the context of mechanisms previously identified in experimental and observational studies (Wang et al., 2023, Cheesman et al., 2024,

Duque et al., 2023) which strengthens the interpretation of our results and aligns them with established physiological processes.

In response to the reviewer's point about insufficient mechanistic on interaction, we have substantially revised the Discussion to explicitly address these processes. For example, we discuss that ozone can impair photosynthetic capacity, accelerate leaf senescence, and disrupt hormonal signaling, all of which influence phenological transitions (e.g., advanced EOS). We also discuss how ozone effects are mediated by stomatal uptake, species-specific sensitivity, background nutrient conditions, and environmental stressors such as drought or heat. These nonlinear interactions can modulate phenological responses depending on regional context and vegetation type. We have included references to controlled-environment and field-based studies that demonstrate these pathways, helping to connect our large-scale remote sensing observations to well-established mechanistic frameworks (see revised Discussion, lines 381-411).

Regarding regional variation, a key objective of our study was to compare ozone-phenology relationships across China, Europe, and the United States, each characterized by different ozone regimes, vegetation compositions, and climatic contexts. We have expanded this comparison in the revised manuscript and now include a more detailed discussion of how regional differences in vegetation composition, meteorology, and pollutant mixtures influence ozone uptake and phenological outcomes. These revisions are intended to strengthen the ecological interpretation of the cross-continental patterns we observed (lines 450-461).

To address the final point on vegetation type difference, we have added an additional analysis on ozone effects on vegetation phenology and greenness across different land cover categories (Figs. S9 and S10). Our results demonstrate consistent patterns across major terrestrial ecosystems, including Deciduous Broadleaf Forests, Mixed Forests, Savannas, and Urban Vegetation, highlighting the broad-scale implications of surface ozone pollution for ecosystem functioning.

Cheesman, A. W., Brown, F., Artaxo, P., et al. (2024). Reduced productivity and carbon drawdown of tropical forests from ground-level ozone exposure. *Nature Geoscience*, 17(12), 1003–1007. https://doi.org/10.1038/s41561-024-01530-1

Wang, Q., Huang, M., Wang, S., Chen, B., Liu, Z., Wang, Z., Chen, S., Li, H., Zhu, T., Li, D., Li, Y., Lin, H., & Sun, L. (2023). Evaluation of the impacts of ozone on the vegetation productivity of woodland and grassland ecosystems in China. *Ecological Modelling*, 483, 110426. https://doi.org/10.1016/j.ecolmodel.2023.110426

Duque, L., Poelman, E. H., & Steffan-Dewenter, I. (2021). Effects of ozone stress on flowering phenology, plant-pollinator interactions and plant reproductive success. *Environmental Pollution*, 272, 115953. https://doi.org/10.1016/j.envpol.2020.115953

Reviewer #2 (Remarks to the Author):

This paper has the noteworthy results of showing, through a partial correlation analyses, the influence of tropospheric ozone levels on continental scale trends in vegetation growing season

parameters as well as integrated growing season and peak vegetation productivity. The hypothesis posed is that ozone levels will delay start of season, while advancing senescence or end of growing season, yielding an overall shortening of the growing season and decline in vegetation productivity. Many local scale studies have also shown similar negative impacts due to surface ozone over specific areas, but this is a first study to look at this across 3 continents and the authors use of partial correlation analyses seeks to separate the ozone influence from that of CO2 concentrations and warming temperatures. There are some apparent weaknesses which may require further analysis and evidence confirm and better understand the results - in order for the study to be convincing.

Response: Thanks for your comments and for recognizing the significance of our study. We have addressed your comments point by point in the following sections, providing additional analyses and clarifications to strengthen our conclusions.

1. Only three factors (temperature, CO2, ozone) on phenology and productivity are analysed. These are the 2 dominant climate change drivers of temperature increases and CO2 increases, with ozone added as the third driver of interest. However, there are other climatic drivers known to affect phenology, in particular precipitation/ water availability and VPD which are ignored throughout the study. Water availability, in particular, is likely the dominant driver of phenology in some regions (e.g. arid US, Mediterranean Europe). Other non-climatic factors such as nitrogen deposition, PM2.5 and pollutants (in China) are also not taken into account. By ignoring these factors, there is a risk that changes in phenology are mis-attributed to ozone effects.

Response: We appreciate the reviewer's suggestion, which has helped us to broaden the scope of our analysis and clarify the limitations of data availability for non-climatic covariates. In the revised manuscript, we have incorporated both precipitation and VPD as additional covariates, as suggested, in the partial correlation and machine learning analyses to account for water availability effects. The updated results confirm that ozone maintains statistically significant impacts on vegetation phenology and productivity, even after controlling these variables, both at spatial and temporal scales. These results are consistent with our original findings, suggesting that the observed ozone-phenology relationships are not an artifact of omitted variable bias. Revised content can be found in 'Results' section (lines 194-197, and lines 239-260) and model specifications are detailed in the updated Methods section (lines 782-791) in the revised version.

We acknowledge the relevance of additional non-climatic factors such as nitrogen deposition, $PM_{2.5}$, and other pollutants, particularly in China. Unfortunately, consistent, high-resolution, national-scale datasets for these variables are not currently available for the entire study period or across all three continents. As such, we could not include them in the quantitative analysis without introducing regional or temporal bias. However, we have now added a paragraph in the Discussion (lines 510-559) explicitly addressing these limitations and outlining how such factors may interact with ozone and other drivers to influence vegetation dynamics.

2. Although the US and Europe had 3-fold lower ozone levels than China, ozone in US/ EU appeared to result in stronger effects on vegetation than China. At least this is based on the strengths of partial correlation analyses, where we see stronger delays in SOS and advances in EOS; as well stronger negative correlations with peak and integrated satellite EVI at the USA/ EU sites relative to China. Maybe a fixed set of latitudes are needed?

Response: Thanks for your comments. We have revised the relevant figures to include information latitudinal changes of the analyzed variables (Figs. 2-3, and S2-S5, S12, and S17-S18).
In our updated results (Fig. S6), the magnitude of ozone effects on SOS, EVImax, and EVIarea is the largest in the U.S., then in China, and the smallest in Europe, while China exhibits the strongest ozone effect on EOS. These revised results align with ecological expectations and address the concern regarding regional comparability. Importantly, we emphasize that the strength of partial correlations reflects not the absolute magnitude of ozone exposure but rather the sensitivity of the local ecosystems to ozone effect, which may differ between continents. We also added discussion on potential ecological and atmospheric mechanisms contributing to regional differences in ozone effects, including differences in background chemistry, vegetation traits, and co-occurring stressors (Discussion, lines 450-461).

3. In addition to the partial correlation analyses of trends, the authors could have provided indications of the slope of the trends, to show, for example, the delay in SOS days/ decade attributed to ozone and quantify the offset to phenology attributed to temperature in each continent. It is possible the time series analyzed, 8-10 years, is insufficient to draw these conclusions. However, the authors could have used the equivalent dataset and products derived from MODIS satellite sensor and have a 24+ years time series results of changing phenologies. It's also curious why the current VIIRS satellite analyses uses different years for China (2015-22) than the other continents (2013-2022).
Response: Thanks for your comments. We have presented the time series and trends of environmental drivers and four vegetation indices for all three regions (Figs. S13–S16). When these figures are considered together with the partial correlation strengths (Fig. 2-3), they allow us to infer for example the delay in SOS days/ decade attributed to ozone. However, we did not directly quantify this, as the relatively short time series precludes robust estimation of long-term trend magnitudes.

The study period was constrained primarily by the availability of reliable, spatially extensive ground-based surface ozone measurements. In China, nationally gridded surface ozone data based on in situ observations are only available from 2015 onwards. Earlier ozone records, where available, are sparse, inconsistent, or localized, making it infeasible to construct a robust spatiotemporal dataset for national-scale phenology-ozone analyses. The comparability of the data is affected by methodological inconsistencies in collection and processing prior to 2013 in Europe and U.S.A. Subsequent data adhere to a more harmonized and rigorous standard, significantly improving their robustness for cross-national analysis. We conducted an extensive review of alternative ozone products, including reanalysis and model-based estimates (e.g., Zhu et al., 2023 and Liu et al., 2022), but found they either lacked observational grounding or did not provide adequate spatial resolution for our purpose of study. We added MODIS phenology data to show robustness of the results, but we couldn't expand it to 24+ years time series due to the ozone data.

While the shorter time series presents limitations for detecting long-term trends, the focus of our study was not on multi-decadal trend estimation, but rather on evaluating spatially explicit relationships between interannual ozone variability and vegetation phenology after controlling for key covariates (e.g., temperature, CO_2, VPD, precipitation). To mitigate the limitations of relatively

short time series and address other comments, we revised our approach to leverage machine learning techniques (see details in the response #2), emphasizing spatiotemporal comparison of regional differences, which reduce the impact of sample size constraints on the results. We also cross-checked results across different satellite products and vegetation types. The statistical significance of our findings and the consistency across sensors support the robustness of our conclusions regarding ozone effects on vegetation phenology.

Zhu, S., Xu, J., Zeng, J. et al. LESO: A ten-year ensemble of satellite-derived intercontinental hourly surface ozone concentrations. *Scientific Data* 10, 741 (2023). https://doi.org/10.1038/s41597-023-02656-4.

Liu, X., Zhu, Y., Xue, L., Desai, A. R., and Wang, H.: Cluster-Enhanced Ensemble Learning for Mapping Global Monthly Surface Ozone From 2003 to 2019, *Geophysical Research Letters*, 49, e2022GL097947, https://doi.org/10.1029/2022GL097947, 2022.

4. Ozone patterns vary highly across latitudes (showing mid-latitude peaks), yet in all figures and discussion, latitude information is missing. In particular Fig. 2 lacks both scale and locational information, and the 3 continents are displayed side by side, as if they are latitudinally aligned, which they are not. It would be very informative to additionally compare results within aligned latitudinal bands.

Response: Thanks for your comments. We have updated all maps to include geographic coordinates (latitude and longitude labels) to improve spatial reference. In addition, we have added line plots showing the latitudinal gradients for the studied variables for all figures. For Figure 2, we now display the three continents within a single global projection to avoid the visual impression that they are latitudinally aligned, and we show the regional specific pattern in Fig. S1.

5. Aside, from latitude, ozone effects are highly variable across sites (Figure 3). The existing background variability would need to be explored in more detail. For example. what factors cause such a spatially variable response to ozone as shown in Figure 3? How are these effects and their uncertainties handled from a statistical point of view. For example, in line 168 the ozone effects on SOS are given as 0.09 +/- 0.02, which implies a high certainty of the effect being positive, which seems to contradict the highly variable effects shown in Figure 3a. The uncertainty metric is given as S.D. in line 150, yet it is clear that S.D. is higher than 0.02 in Figure 3a. As such, the confidence in the results seem to be overstated.

Response: Thanks for your comments. We have updated all results after incorporating additional covariates (VPD and precipitation). In the revised analysis, we find that the average $R_{AOT40–SOS}$ relationship is positive (0.06 ± 0.004), indicating that while the overall temporal effect of ozone on SOS is weakly positive, it exhibits substantial spatial variation across sites. This high variability is also evident in Fig. 3a and reflects differences in vegetation types, climate and environmental conditions, and local pollution regimes. Such heterogeneity is expected and not surprising given the complex ecological and atmospheric processes involved.

To address the reviewer’s concern about uncertainty reporting: in the original version we used standard deviation (SD), which characterizes the dispersion of site-level responses. However, this created confusion when interpreting the precision of the mean regional effect. In the revised version,

we report standard error (SE) instead of SD when presenting average relationships (e.g., 0.06 ± 0.004). SE more appropriately reflects the uncertainty in estimating the mean effect size across all sites, whereas SD describes the spread of individual site-level responses. The small SE indicates that, despite high variability at local scales, the mean regional effect is estimated with high precision. Additionally, we conducted a new analysis stratified by land cover type (Figs. S9 and S10). While the direction of ozone effects is generally consistent across land covers, their magnitudes vary considerably, further supporting the interpretation that ecosystem traits and environmental context modulate ozone sensitivity.

We also added discussion of potential drivers of site-level variability, including species composition, background atmospheric chemistry (see lines 412-419 and 450-461 in discussion). Although comprehensive data on these drivers are not available at national scales, we highlight them as key factors contributing to the observed heterogeneity in the discuss.

6. It would add much value to this study to further select targeted areas that show more explicitly phenology change patterns (as in Figure 1) across the sources of variability, including vegetation type, latitude, urban/ rural classes, etc. Its' quite difficult to follow the authors observed interpretation from very small global maps.

Response: Thanks for your comments. We agree that a more detailed spatial and categorical analysis strengthens the interpretation of our findings, particularly given the inherent variability in ozone-vegetation interactions. As suggested, we have conducted additional stratified analyses to explicitly examine how phenological and productivity responses to ozone vary across different vegetation types and urban/rural settings (Figs. S9-10). This analysis revealed that 77% of the sites are situated in urban areas (thus representing urban and peri-urban vegetation), while 23% are located in forests (Fig. S9). We consistently found that elevated ozone levels are associated with a shortened growing season and reduced vegetation productivity across both urban and forested sites. To improve spatial interpretability, we have generated higher-resolution regional maps for the three studied region (Fig. S1) that more clearly illustrate spatial clusters of ozone level. We also added latitudinal gradient plots (zonal means) of both phenological metrics and ozone concentrations in relevant figures (Figs. 2, 3, S2-5, S12, and S17-18), which help highlight large-scale spatial patterns and dependencies that were difficult to discern in the global maps alone.

7. The future projections are of purely statistical nature and assume that temperature and CO2 effects in the future are the same as they are in the past. This is a strong assumption as effects are non-linear (e.g. biological temperature responses show an optimum, CO2 effects saturate). In addition, other climate change factors, in particular water and nutrient dynamics, are ignored. I suggest running a process-based model such as CLM into the future. This would allow to a) isolate ozone effects and b) take all major climate change factors and their non-linearities into account.

Response: Thanks for your comments. To our knowledge, current process-based modeling approaches like CLM do not yet include an explicit representation of ozone effects on vegetation phenology, particularly the observed delays in SOS and advances in EOS reported in our study. While CLM does simulate ozone impacts on photosynthesis and stomatal conductance, these effects are not yet linked to phenological transitions, which are typically parameterized as functions of temperature and photoperiod. As such, CLM and other land surface models are presently not

equipped to isolate or project ozone–phenology interactions, which is a core focus of our study. Given this limitation, we updated our originally multiple linear regression model with a data-driven, machine learning framework that enables us to quantify and project nonlinear ozone's effects on phenology while controlling for other variables such as temperature, CO_2, VPD, and precipitation. This allows us to address part (a) of the reviewer's suggestion by quantifying ozone-specific effects in a nonlinear framework. For future projections, we further improved our approach by replacing the earlier simplified fixed-rate scenarios with spatially explicit, scenario-dependent ozone projections simulated using the Community Earth System Model version 2 (CESM2) (Bhattarai et al. 2024). These projections are based on CMIP6 anthropogenic and biomass burning emissions, with land use, greenhouse gases, and short-lived species prescribed under different Shared Socioeconomic Pathways (SSPs). They account for region-specific ozone responses, nonlinear chemical behavior, and sensitivity to changing emission baselines.

In our revised manuscript, we have added a discussion highlighting that current versions of CLM do not account for ozone effects on vegetation phenology and productivity (Lines 489-503). Our findings demonstrate clear ozone impacts, suggesting this mechanism should be incorporated in future model developments to improve their completeness and accuracy.

Bhattarai, H., Tai, A. P. K., Val Martin, M., and Yung, D. H. Y.: Impacts of changes in climate, land use, and emissions on global ozone air quality by mid-21st century following selected Shared Socioeconomic Pathways, Science of The Total Environment, 906, 167759, https://doi.org/10.1016/j.scitotenv.2023.167759, 2024.

Detailed comments:

Abstract:

- The statement in Abstract "Using multiple ground and satellite observations", could be misinterpreted as several ground and satellite datasets. More accurately, it would be clearer to write "Using ground observations of ozone and satellite observations".

Response: Thanks for your comments. We have revised the corresponding content.

- The motivation for this study needs to be clearer (Lines 23-24). Is the gap in knowledge that ozone effects on vegetation phenology are unknown or is it that large scale ozone effects are unknown?

Response: Thanks for your comments. The knowledge gap that our study answers is that large-scale ozone effects are not well understood. We have revised the corresponding content.

Results:

Figure 2a-c: time period of observations should be given in Figure caption.

Response: We have added time period.

Figure 2d: maps are very small and difficult to read

Response: We have revised the original Figure 2 to Fig. 2 and Fig. S1.

Most Figures- missing scale and latitude/long information.

Response: We have added scale and latitude/long information in these relevant figures.

Figure 2d results (line 152). Authors write "This indicates elevated ozone is associated with the lengthening of growing season by shifting both spring and autumn phenology." This runs counter to the rest of the study.

Response: We have corrected it.

Reviewer #3 (Remarks to the Author):

I co-reviewed this manuscript with one of the reviewers who provided the listed reports. This is part of the Nature Communications initiative to facilitate training in peer review and to provide appropriate recognition for Early Career Researchers who co-review manuscripts.

Response: Thanks for your comments.

Reviewer #4 (Remarks to the Author):

The paper "Ozone mitigates extended growing season and enhanced vegetation greenness driven by climate change" by Yin et al submitted to Nature Communications investigates the influence of increasing surface ozone concentration on vegetation phenology and greenness. Main results reported indicate that higher ozone concentration will mitigate climate change impacts expected to lengthen the growing season by decreasing the season up to 10 days and also reducing the accumulated enhanced vegetation index. The paper reads well and has a clear message, that brings the management of surface ozone concentration to an urgent level. My review focused on the methods used for the analyses. The authors indicate they have done a "partial correlation analyses", which is an interesting method to use in this case aiming to tease out the effect of the variables of interest (e.g., ozone) by controlling the effect of other variables (e.g., temperature, in this case). There are a few points around the methods and analyses used that would be good to have clarified in the manuscript:

Response: Thanks for your constructive comments. We addressed your comments point by point in the following content.

1) Could the authors confirm if equation 2 is correctly written? Shouldn't the second part of the numerator be "- R(X,Z) R(Y,Z)" instead of "- R(X,Y) R(Y,Z)"?

Response: Thanks for your comments. We have corrected this mistake in the revised version.

2) More detail on the difference of the models for the spatial and temporal components would be useful, for example, how did the authors deal with correlation between the variables that were not being controlled for in each of these analyses?

Response: Thanks for your comments. In our updated version, we employed three analytical approaches to rigorously address variable correlations, and we clarify how correlations among variables were addressed in both the spatial and temporal analyses: (1) Partial correlation analysis was used to isolate ozone effects while controlling for key confounding variables (temperature, CO_2, and now additionally humidity and VPD in the revised version) spatially (Fig. 2) and temporally (Fig. 3); (2) a LightGBM machine-learning model was developed to predict phenological metrics while handling multicollinearity through gradient boosting structure and automated feature selection. After rigorously validating the accuracy and stability of its performance, we utilized the trained model to project future phenological metrics under varying scenarios; and (3) SHAP analysis was

applied to quantify variable contributions and interactions. Together, these approaches ensure that correlations are explicitly addressed and that spatial and temporal ozone–phenology relationships are assessed in a consistent, robust framework. We also acknowledge that additional non-climatic factors (e.g., N deposition, $PM_{2.5}$) could not be included due to data limitations, which we discuss in the revised text (lines 504-559).

3) The phenological prediction was based on MLR, which includes the same assumptions as simple linear models, however none of the checks on these assumptions was reported in the manuscript. Adding a summary of these checks would be beneficial to aid interpretation of the model results.

Response: Thanks for your comments. In our revised manuscript, we have replaced MLR approach with a LightGBM machine learning framework, which offers distinct advantages for our analysis: (1) it inherently handles nonlinear relationships between predictors and phenological responses, (2) it is robust to multicollinearity among environmental variables through its tree-based learning structure, unlike MLR assuming linearity and independence among predictors, and (3) it automatically captures interaction effects. Furthermore, its built-in feature selection mechanism reduces the impact of highly correlated variables by prioritizing splits that minimize loss, thus enhancing interpretability and predictive accuracy. We also employed a rigorous 10-fold cross-validation approach to evaluate the LightGBM model's performance, which provides robust assessment of predictive accuracy while efficiently utilizing all available data. The cross-validation results demonstrated consistent predictive performance across all folds, indicating the reliability and robust of our model. Furthermore, we employed SHAP analysis to provide interpretable, quantitative measures of variable importance that address the reviewer's concern about result interpretation. We clarify the model description and method in the revised manuscript lines 793-833.

4) The authors present equation 3 for the MLR stating it represents a linear combination of multiple independent variables, however they never confirm independence of the variables included as predictors in this model. For example, are temperatures for SOS and EOV correlated? OR the EVImax with EVIarea? A statement about the correlations (or a table with the results in SI) could be beneficial.

Response: Thank you for raising this point. In the revised manuscript, we explicitly address correlations among predictors by using LightGBM with SHAP, which can account for nonlinear and interactive relationships between environmental drivers and phenological metrics (see response #3 for details). To further avoid confounding, we developed four separate LightGBM models for SOS, EOS, EVImax, and EVIarea, each with its own predictor set. For example, temperature used for SOS prediction is not included in the EOS model, and EVI_{max} is not used to predict EVI_{area}. This separation ensures that correlations between phenological metrics do not bias model results, and we clarify this in the revised text.

5) The prediction into the future is likely to be the result of extrapolation but no information is provided in the Methods. Although it is clearly a problem for the future scenarios and also the reason why multiple scenarios are tested, it would still be good have an idea of how far the variables used as predictors are from the ranges of values used in the training models.

Response: Thanks for your comments. In the original version of the study, we designed idealized ozone scenarios with multiple trends (e.g., increasing, stable, decreasing) to explore the sensitivity

of phenological responses to ozone changes. However, in the revised manuscript, we replaced those simplified assumptions with spatially explicit, scenario-based projections of ozone concentrations derived from the CMIP6 SSP5–8.5 scenario, as published by Bhattarai et al. (2024). It's a more robust representation of ozone changes under greenhouse gas forcing by accounting for nonlinear interactions between ozone precursors and climate feedbacks. The ozone dataset under future scenario was simulated by the Community Earth System Model version 2.1.3 (CESM2.1.3), employing its atmospheric component CAM4-chem, which incorporates full tropospheric O_3-NO_x-CO-VOC-aerosol chemistry based on the MOZART-4 mechanism. The model was run in time-slice mode at ~1° horizontal resolution (0.9° latitude × 1.25° longitude) with 26 vertical levels extending to ~40 km. Land–atmosphere processes were represented by the Community Land Model version 5 (CLM5) with active biogeochemistry, in which biogenic VOC emissions were calculated online using MEGANv2.1 as a function of vegetation type, meteorology, and CO_2 levels, while dry deposition was dynamically linked to stomatal conductance. Anthropogenic and biomass burning emissions followed the CMIP6 inventory, while future scenarios of land use, greenhouse gases, and short-lived species were prescribed from Shared Socioeconomic Pathways (SSPs). The detailed of the ozone dataset can be found in the section "Future Ozone Scenarios" (lines 708-735). We clarify the model description and method in the revised manuscript lines 793-833.

6) A statement clearly discussing the results for the predictors across scenarios should clearly explain the results in different directions and in which cases they happen (e.g., many of the figures in SI show increasing and decreasing trends, however the manuscript focus on a single trend: ozone concentrations will reduce the expected effects of climate change.

Response: Thanks for your comments. Please see our response #5 above.

REVIEWERS' COMMENTS

Reviewer #1 (Remarks to the Author):

The paper has been revised in accordance with the referees' comments, incorporating additional satellite data and statistical methods (e.g., LightGBM). I have no further comments and agree with the acceptance of the study.

Response: Thanks for your positive comments. We are very grateful for your positive feedback on our revisions, and we appreciate your suggestions, which have greatly improved our manuscript.

Reviewer #2 (Remarks to the Author):

My ECR co-reviewer and myself wish to acknowledge the significant revisions and new analyses conducted by the authors which have resulted in a much improved manuscript with results that are very convincing. The authors have now included significant co-variates into their partial correlation analyses, which have maintained the initial statistically relevant ozone impacts in both the spatial and temporal domains. The few variables that could not be integrated into the analyses, such N-deposition, were properly acknowledged as limitations in the Discussion section, making this section more interesting for future studies/ thoughts.

We are also now satisfied with the uncertainty analyses and other responses to our comments.

Response: Thanks for your previous suggestions and for your positive comments.

In fact, the only remaining concern is around the response to our comment #6 that focuses on more detailed targeted areas to confirm findings. The paper is overall written in the style of a global analyses on vegetation phenology, however, we feel this may be quite misleading and lead to misinterpretations by many, since as we know most ozone networks and sparsely located and prioritize urban and peri-urban locations only because population exposure is highest there and thus most relevant for health risk assessments.

The authors mention that more ground ozone measures are needed in rural areas, however, in the authors response to our comment 6, it was pointed out that 77% of the sites used in this paper were urban sites. Of the remaining 23%, the majority of these are most likely very close to urban centers? The spatial cluster maps give the impression and exaggerated wider coverage. The authors do mention that more sites outside urban areas are needed, but only at the end of the Discussion. We feel this is needs to be stated upfront and probably in the abstract. It does not deter from the significant findings of this study - it merely constrains the results to where they belong - urban vegetation and their adjacent ecosystem surroundings. The authors could have selected the 'most' remote ground ozone sites to compare against the urban sites, if they really wanted to claim a global analyses - or used satellites. We think urban vegetation also has different temperature and CO_2 regimes, in contrast to rural and remote areas.

Response: Thanks for your comments. In the revised manuscript, we now clarify this point explicitly at the beginning of the study description in the Introduction. We have also retained and strengthened the discussion of this limitation, emphasizing the need for expanded rural monitoring to improve spatial representativeness. Our intention is not to overstate the spatial coverage as fully global, but to provide a large scale, multi regional assessment based on the best available long term ground observations. We believe the revised text now makes this constraint clear upfront and reduces the risk of misinterpretation.

"We quantify ozone exposure using Accumulated dose of ozone Over a Threshold of 40 ppbv (AOT40) and average surface ozone concentration derived from ground-based observation networks, most of which are located in urban areas." (Lines 99-101)

"Because most monitoring sites are in urban areas, our estimates of ozone effects primarily capture responses of urban vegetation, while still providing insight into ozone–phenology relationships across broader land cover types." (Lines 108-110)

The authors did mention satellite-based ozone products in Discussion and noted their limitations. However, it could make better discussion to say ground-calibrated ozone fused with satellite ozone can enable spatial extrapolations and provide the means to assess phenology- ozone relationships distant from urban areas (this is done with EC flux towers and satellites). Many studies are being published with ozone and smoke from remote fires. Current satellites, as TROPOMI, provide global, repeated coverage, making it possible to track broad changes in tropospheric ozone that are not feasible with sparse ground-based networks alone. This makes for more interesting,, positive discussions (we're not asking for more analyses).
Response: Thank you for this constructive suggestion. We have incorporated these into the discussion. These additions aim to provide a more forward looking and balanced perspective on how integrated observation systems can strengthen future large scale assessments.

"At the same time, satellite instruments such as TROPOMI now provide global, repeated coverage of tropospheric ozone, enabling the detection of broad spatial and temporal patterns that cannot be resolved by sparse ground networks alone. Although satellite retrievals have limitations and require careful validation, fusing satellite products with ground calibrated observations offers a promising pathway for spatial extrapolation of ozone estimates beyond urban cores. Similar data fusion approaches have been successfully applied in eddy covariance flux studies to extend site level measurements to regional scales, and they are increasingly used to track ozone and smoke from remote fires" (Lines 304-312)

Minor comments: the authors should provide actual url links to the ozone sites/ data, rather than links to the agencies. I click on the US- EPA ground ozone link and it took be to the homepage of EPA. I searched for ozone monitoring and it gave 500,000 sites?
Response: Thanks for your comments. We have updated the URL in the revised manuscript and in the Data Availability Statement. In addition, we have clarified in the Methods section that the surface ozone observation data used in this study was selected based on our criteria, so it includes fewer sites. Please see details in Lines 360-375.

Reviewer #3 (Remarks to the Author):

I co-reviewed this manuscript with one of the reviewers who provided the listed reports. This is part of the Nature Communications initiative to facilitate training in peer review and to provide appropriate recognition for Early Career Researchers who co-review manuscripts.
Response: Thank you.

Reviewer #4 (Remarks to the Author):

The authors have done a huge amount of work in the revision of this manuscript with many additions alleviating most of the concerns in the comments I provided. I particularly liked the addition of LightGBM and SHAP. My comments were therefore mostly addressed. The only point missing is a quick visual representation of the differences in the data used for model development and for prediction into the future (e.g., boxplots showing how much the future data differs from the data used to train the model). Together with a brief comment on the results, this representation would assist assessing the level of extrapolation included in the results.

Response: Thanks for your positive comments. We added Fig. S26 showing the range of input variables for the model in training versus prediction datasets. Comparing the distribution ranges of the variables between the training data and future scenarios, we found that the values of meteorological factors (VPD, temperature, and precipitation) under future scenarios are entirely within the range of the model training data, indicating that the model is not extrapolating beyond its learned climate variability for these drivers. Although ozone and CO2 concentrations increase substantially under future scenarios, this pattern is expected and reflects the assumption of the future scenarios SSP585. Overall, these results indicate that the projections involve minimal extrapolation for climate variables, while changes in atmospheric composition follow the modeled scenario assumptions. Please see details in Lines 582-586.
